# Engineered *Lactobacillus reuteri* for scavenging reactive oxygen species and modulating oral microflora in periodontitis therapy

**DOI:** 10.1038/s41368-025-00418-z

**Published:** 2026-02-10

**Authors:** Yuqiang Wang, Ying Tang, Qianxiao Huang, Jiaxin An, Yueli Zhou, Hongye Yang, Fangfang Song, Xianzheng Zhang, Cui Huang

**Affiliations:** 1 State Key Laboratory of Oral & Maxillofacial Reconstruction and Regeneration & Key Laboratory of Oral Biomedicine Ministry of Education & Hubei Key Laboratory of Stomatology & School & Hospital of Stomatology, Wuhan University, Wuhan, China; 2 Key Laboratory of Biomedical Polymers of Ministry of Education & Department of Chemistry, and Institute for Advanced Studies, Wuhan University, Wuhan, China

**Keywords:** Microbiology techniques, Periodontitis

## Abstract

The onset and progression of periodontitis are closely associated with subgingival dysbiosis and excessive localized oxidative stress. While some oral probiotics exhibit certain inhibitory effects on periodontitis-related pathogens, they often struggle to effectively colonize and antagonize these pathogens due to the complex oxidative stress at the site of periodontitis. In this study, we engineer *Lactobacillus reuteri* with a reactive oxygen species (ROS)-responsive adhesive polymer (phenylboric acid-dopamine-hyaluronic acid) (LR@PDH). In the periodontitis microenvironment, this polymer can consume ROS and then expose the phenolic hydroxyl group of dopamine, promoting the selective adhesion and colonization of *Lactobacillus reuteri* at the site of inflammation to antagonize pathogens. The results show that, compared to conventional probiotic therapy, inflammation-responsive adhesive *Lactobacillus reuteri* effectively alleviates local oxidative stress, reduces the abundance of pathogenic bacteria in the subgingival microbiome, and inhibits the progression of periodontitis. Additionally, its good biocompatibility and safety highlight its potential as a therapeutic approach for clinical treatment of periodontitis.

## Introduction

Periodontitis, a chronic bacterial infectious disease, has influenced over 1.09 billion people around the world as one of the most common oral diseases^1,2^. The pathogenesis and progression of periodontitis are strongly associated with dysbiosis of the subgingival microbiota^3,4^. In the periodontal inflammation, subgingival plaque undergoes a marked increase in biomass and a significant shift in composition, characterized by a notable rise in pathogenic Gram-negative bacteria such as *Porphyromonas gingivalis* (*P. gingivalis*) and *Fusobacterium nucleatum* (*F. nucleatum*)^5^. This microbiota dysbiosis continuously stimulates periodontal tissue, triggers an excessive host immune-inflammatory response, which leads to the destruction of periodontal tissues^6–8^. During the excessive immune response, neutrophils and other immune cells generate reactive oxygen species (ROS) through the respiratory burst^9^, leading to lipid peroxidation, protein oxidation, and DNA damage. This process activates inflammatory, apoptotic, and autophagic pathways, including NF-κB and JNK, further compromising periodontal tissue integrity^10^. Studies have shown that oxidative stress levels are significantly elevated in periodontitis patients compared to individuals with gingivitis or healthy controls, highlighting a strong correlation between oxidative stress and periodontitis progression^11^. Consequently, strategies focused on restoring microbial homeostasis, reducing ROS accumulation, and controlling oxidative stress and inflammation have become critical research priorities^12,13^.

Probiotics therapy has emerged as a promising therapeutic approach for modulating microbiota dysbiosis, with applications in gastrointestinal disorders, tumors, ischemic cardiac injury, and periodontitis^14–17^. Recently, several material-assisted probiotic strategies have been developed for the treatment of periodontitis^18,19^. An injectable blue-light–responsive hydrogel incorporating *L. rhamnosus* GG (LGG) (LRG-P@LGG) has been designed to achieve comprehensive periodontal therapy through antibacterial, anti-inflammatory, and tissue-regenerative functions^19^. In addition, puerarin-modified live *L. rhamnosus* was combined with chitosan and hyaluronic acid to prepare probiotic nanoparticles, which were subsequently encapsulated in hydrogel microspheres to modulate the microbiota and promoting immune restoration^18^. Studies have demonstrated that *Lactobacillus reuteri* (*L. reuteri*) can reduce the proportion of periodontal pathogens, such as *P. gingivalis* and *F. nucleatum*, in the subgingival microbiota through mechanisms such as competition for nutrients and secretion of bioactive compounds^20,21^. However, the challenging microenvironment of oral diseases, including oxidative stress at the site of periodontitis, compromises probiotic viability and retention, limiting the effectiveness of traditional therapies like *L. reuteri* therapy in the hostile environment^22,23^. To address these challenges, enhancing the retention and functionality of probiotics at inflammatory sites is essential for sustained modulation of microbiota dysbiosis^24^. Recent advancements suggested that, compared to single probiotic treatments, the strategy of combining probiotics with polymers, nanomaterials, or drugs can enhance the therapeutic efficacy of probiotics in challenging environments, providing valuable insights for future therapeutic approaches^25,26^.

**Fig. 1:**
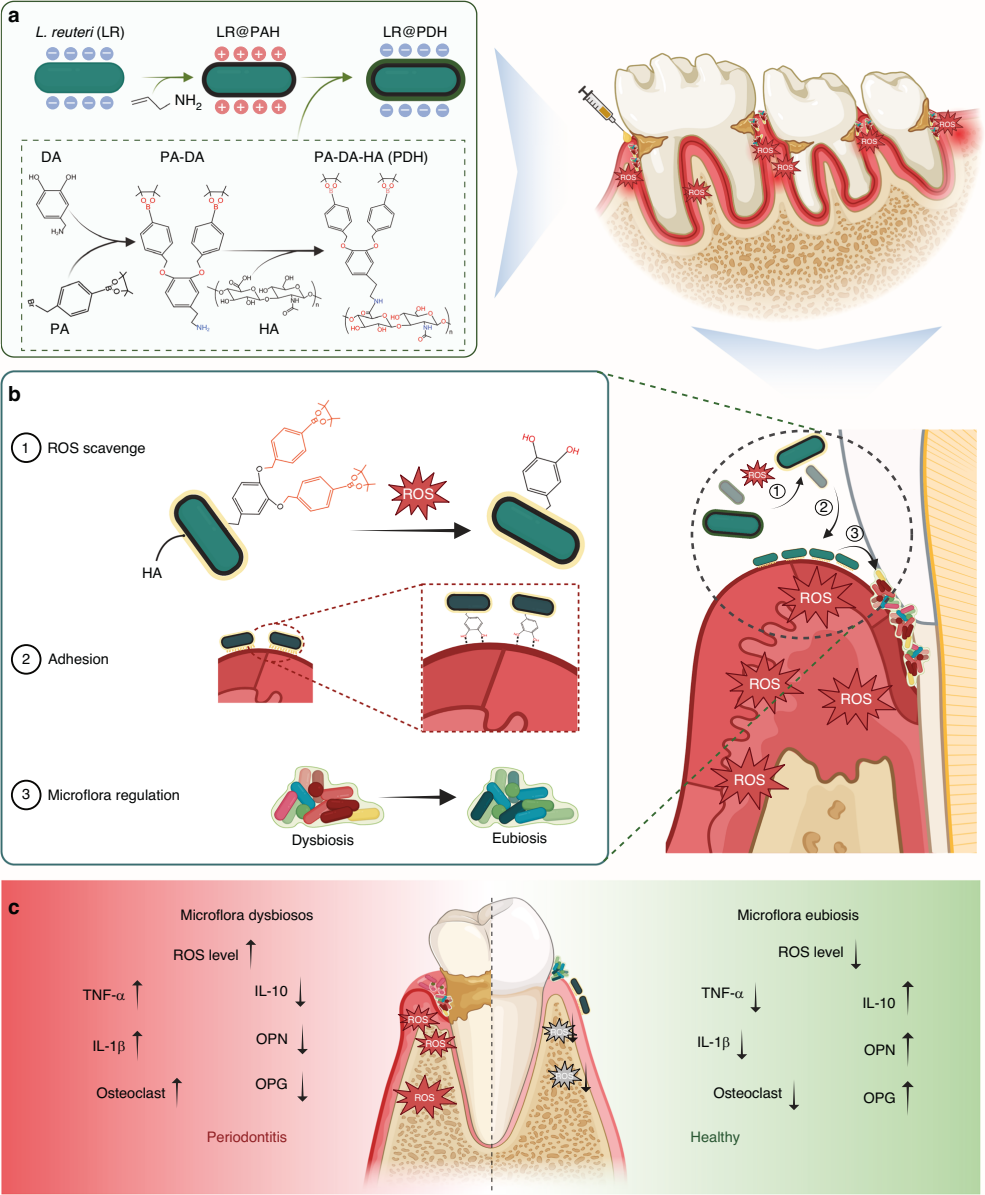
Schematic illustration of the preparation of LR@PDH and its mechanism for periodontitis treatment. **a** Illustration of the procedure for preparation of LR@PDH. **b** Illumination of LR@PDH ROS-scavenging, adhesion, and microflora regulation to the periodontitis. **c** Illumination of LR@PDH regulation of periodontal inflammatory environment. Created in https://BioRender.com

Here, to address the limited efficacy of traditional probiotic therapies, we developed an inflammation-responsive polymer (phenylboric acid-dopamine-hyaluronic acid)-modified *Lactobacillus reuteri* (LR@PDH). This system can selectively adhere to oral inflammatory sites, scavenge excessive ROS, and continuously modulating periodontal dysbiosis and restoring microbial homeostasis (Fig. 1). Specifically, phenylboric acid, dopamine, and hyaluronic acid are conjugated to form the inflammation-responsive polymer (PDH), which is then coated onto the surface of *L. reuteri*, resulting in the development of an engineered probiotic (LR@PDH) with ROS-scavenging properties and enhanced adhesion. In an oxidative stress environment, ROS induce cleavage of the ester bonds between phenylboric acid and dopamine, reducing ROS levels and exposing the phenolic hydroxyl group of dopamine. These groups facilitate robust adhesion to inflammatory tissues through covalent interactions with amino, sulfhydryl, and imide groups, significantly prolonging probiotic retention at the target site^27^, thereby effectively combating pathogenic bacteria and achieving microflora regulation. In vitro and in vivo experiments validated the therapeutic efficacy of LR@PDH, offering a precise strategy for targeted periodontal therapy. This approach holds promise for advancing periodontitis treatment by addressing the dual challenges of microbial dysbiosis and oxidative stress.

## Results

### ROS levels in periodontitis rats

**Fig. 2:**
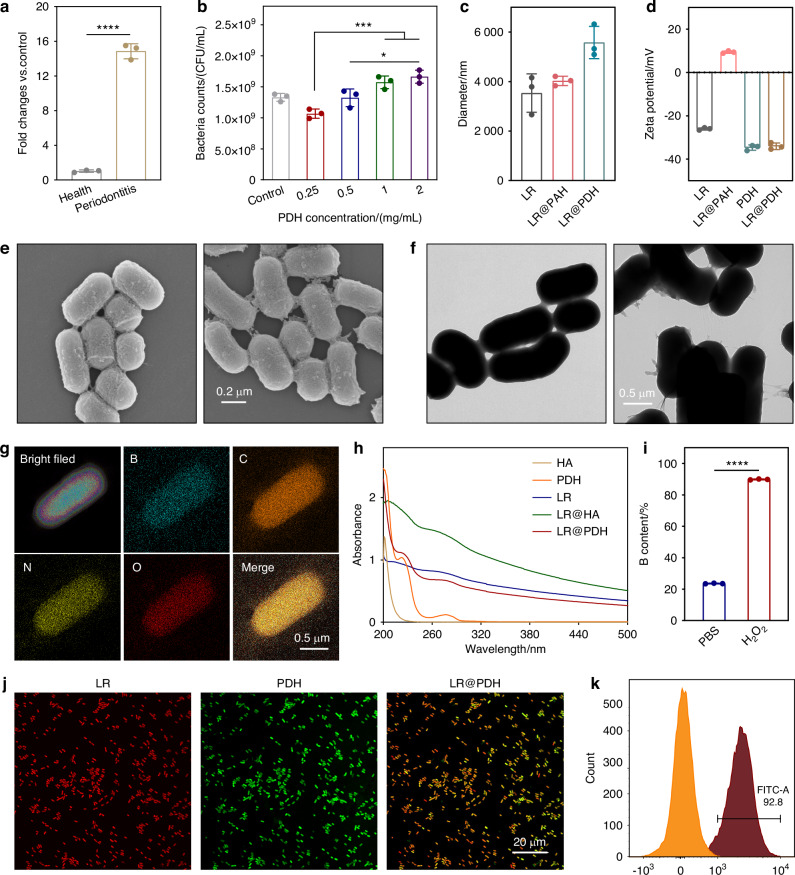
Preparation and characterization of LR@PDH. **a** Changes of ROS levels in gingival crevicular fluid of health and periodontitis rats. **b** Survivals of LR after coating different concentration of PDH. **c** Sizes and **d** zeta potentials of LR, LR@PAH and LR@PDH. **e** SEM and **f** TEM images of LR and LR@PDH. **g** HAADF-STEM images of LR@PDH, and distribution of elements B, C, N, and O. **h** UV spectrum of LR, PDH, HA, LR@HA and LR@PDH. **i** Element B content in the supernatant of LR@PDH with treatment of PBS and H_2_O_2_. **j** CLSM images of LR@PDH (Cy5-labeled LR (red) coated with FITC-labeled PDH (green)). **k** Flow cytometry of LR and LR coated FITC-labeled PDH. ***P < 0.001 and ****P < 0.000 1. The data are presented as mean ± SD, *n* = 3

To investigate the saliva ROS levels of periodontitis rats, we established periodontitis rat model by ligation with silk for 7 days. The ROS levels between healthy and periodontitis rat was semi-quantitative measured by using DCFH-DA fluorescence probe. As shown in Fig. 2a, the results suggested that the gingival crevicular fluid (GCF) ROS levels of periodontitis rats were significantly higher, reaching approximately 14.8-fold higher than those in healthy rats. Manjeu et al. found the reactive oxygen metabolite levels in GCF in generalized chronic periodontitis patients were approximately 3-fold higher than those in the control group^28^. And the ROS levels in GCF potentially affected by severity of inflammation, time point of sample collection and detection method. Excessive ROS can activate inflammatory signaling pathways, such as NF-κB and MAPK, which promote the release of pro-inflammatory cytokines (e.g., IL-1β, TNF-α) and intensify local inflammatory responses^29^. Moreover, oxidative stress induced by ROS can trigger apoptosis in gingival fibroblasts and periodontal ligament cells, thereby weakening the tissue’s repair capacity^30^.

### Preparation and characterization of LR@PDH

Thus, we successfully constructed a ROS-scavenging engineered probiotic system capable of eliminating excessive ROS at periodontal inflammatory sites and specifically adhering to inflamed oral tissues. Through these functions, the engineered probiotics can continuously modulate periodontal dysbiosis and help maintain microbial homeostasis. As illustrated in the Fig. 1, DA (Boc) was firstly generated by introducing Boc group to dopamine to protect the phenolic hydroxyl group from oxidation. The ^1^H NMR spectrum of DA(Boc) showed the characteristic peak of Boc (f) is observed near δ = 1.3 and the area ratio of the characteristic peak of the benzene ring hydrogen to Boc as 1:9 (Fig. S1). Then, the phenolic site of DPA (Boc) was bonded to phenylboronic acid (PA) to form Boc-DA-PA, which could be broken when it encounters ROS. As shown in Fig. S2, the characteristic peak of phenylboronic acid is observed near δ = 1.27, and the area ratio of the characteristic peak of the benzene ring hydrogen, Boc, and phenylboronic acid was 1:9:24, confirming the successful synthesis of PA-DPA(Boc). As exhibited in Fig. S3, the disappearance of the characteristic peak of Boc near δ = 1.3 and the area ratio of the characteristic peak of the benzene ring hydrogen to phenylboronic acid as 1:24, revealing the successful removal of Boc. Subsequently, PA-DA was grafted with hyaluronic acid (HA) to synthesis PA-DA-HA (PDH). Fig. S4 showed the ^1^H NMR spectra of HA and PDH, the characteristic peaks of the benzene ring hydrogen are observed between δ = 6.6 and 7.8, but the characteristic peak of phenylboronic acid is not prominent, likely due to the influence of the hyaluronic acid polymer. However, the spectrum shows that the hyaluronic acid polymer chain has the same characteristic peaks (δ = 6.6–7.8) as the benzene ring of PA-DA, confirming the successful synthesis of PDH.

*L. reuteri* (LR) is one of most commonly used probiotics in dentistry^31^. The peptidoglycan sourced from of *L. reuteri* can inhibit the lipopolysaccharide-induced inflammatory responses of *Porphyromonas gingivalis*^32^. In addition, in supportive periodontal therapy for periodontitis, individual oral administration of *L. reuteri* tablet has been found to exert a positive clinical effect^33^. Therefore, *L. reuteri* (LR) was selected as a model probiotic for construction of engineered probiotic (LR@PDH). To conjugate the PDH onto the surface of LR, poly (allylamine hydrochloride) (PAH) was first employed to load on the surface of LR (LR@PAH) rendering is positive charged, and then PDH was coated on LR@PAH to obtain LR@PDH due to the electrostatic interactions.

It is important to note that electropositive PAH may compromise the integrity of electronegative cell membranes, thereby potentially affecting the bioactivity of LR. To enable the load with appropriate amounts of PAH, different concentrations were adopted to encapsulate LR. As shown in Fig. S5, the viability of LR@PAH was significantly reduced with the elevated concentration of PAH, therefore, 0.125 mg/mL PAH was employed to further study. Subsequent, PDH with a battery of concentrations were applied to construct LR@PDH, and the results revealed that the viability of bacteria was better as the concentration of PDH increased (Fig. 2b and Fig. S6). The positively charged polymer PDH may neutralize the membrane damaging effect of the negatively charged PAH and therefore mitigate the injury to LR. Given this situation, the PDH concentration used to build the engineered probiotics was determined as 2 mg/mL. After coating the LR with PAH and PDH layers, the hydration diameter of LR increased progressively from approximately 3.5 to 4.5 to 5 μm (Fig. 2c), and the zeta potential shifted from about −26 to +9.4 and then to −34 mV (Fig. 2d). Furthermore, additional coating of LR@PAH with hyaluronic acid (HA) layers resulted in a change of zeta potential from +8.9 mV to −7.4 mV (Fig. S7). The images observed by scanning electron microscopy (SEM) and transmission electron microscopy (TEM) suggested a rougher surface from LR@PDH compare with bare LR, providing a fact that LR@PDH was successfully constructed (Fig. 2e and f), and the presence of B element confirmed that LR was successful coated with PDH from the TEM mapping elemental analyses (Fig. 2g). Results from ultraviolet visible spectrum indicated that LR@PDH displayed a characteristic absorption band at approximately 222 nm, corresponding to the PDH component, whereas LR@HA exhibited a peak near 201 nm, characteristic of HA. These findings confirm the successful deposition of PDH and HA layers on the surface of LR (Fig. 2h). Moreover, 200 μmol/L H_2_O_2_ was added in the solution of LR@PDH, after shaking for 1 h the element B in supernatant was measured by an inductively coupled plasma mass spectrometry (ICP-MS), the results showed in Fig. 2i indicated that the phenyl borate group of LR@PDH was fractured and the phenylboric acid were released at the presence of ROS. Furthermore, FITC-labeled PDH layer was co-located with the Cy5-labeled LR by confocal laser scanning microscope (Fig. 2j), and the flow cytometry curve clearly illustrated the higher fluorescence intensities of FITC-labeled LR@PDH compare with bare LR, further confirming the coating of the PDH on the surface of LR (Fig. 2k).

### ROS scavenging ability of LR@PDH

First of all, LR, LR@HA and LR@PDH were respectively incubated in a MRS medium for 12 h to estimate whether the PDH coating affected the growth and proliferation of LR cells. As depicted in Fig. S8, the growth curves of the three groups showed similar growth rate, revealed the PDH coating had no significant impact the growth and proliferation of LR. Subsequently, the metabolic activity of LR, LR@HA, and LR@PDH was monitored at 12-h intervals over a 7-day period (Fig. S9). As illustrated in Fig. S9, consistent with the temporal variation observed for LR, the bacterial viability of LR@HA and LR@PDH started to decline after the third day, and the metabolic activity was nearly completely lost by the sixth day.

**Fig. 3:**
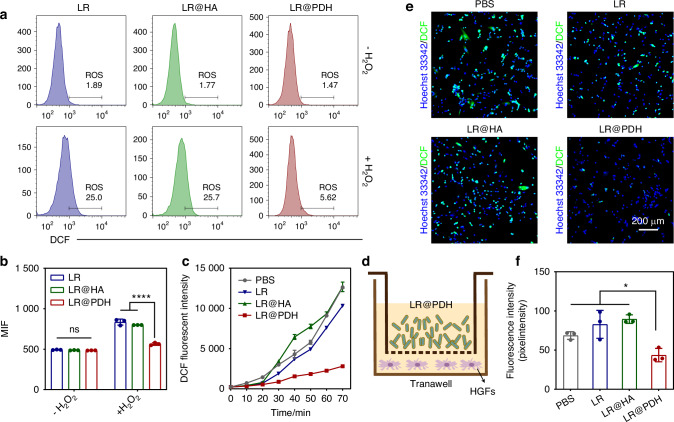
ROS scavenging ability of LR@PDH. **a**, **b** DCF fluorescence intensity within LR, LR@HA and LR@PDH after incubation with H_2_O_2_ were detected by flow cytometry. **c** DCF fluorescence intensity in the supernatant containing 400 μM H_2_O_2_ after treated with PBS, LR, LR@HA and LR@PDH. **d** Schematic of HGFs cells co-cultured with probiotics in the Transwell system. **e** Representative images and **f** quantitative analysis of intracellular ROS levels of HGFs cells treated with 200 μmol/L H_2_O_2_ after co-cultured with PBS, LR, LR@HA and LR@PDH. *P < 0.05 and ****P < 0.000 1. The data are presented as mean ± SD, *n* = 3

Then, the ROS-scavenging activity of LR@PDH was measured by 2,7-dichlorofluorescin diacetate (DCFH-DA), which can be oxidized and the DCF (a fluorescent indicator) was formed when met the ROS. Firstly, the intra-bacterial ROS levels of LR, LR@HA and LR@PDH were evaluated after incubation with H_2_O_2_ treatment. As a result, the ROS levels inside LR@PDH were significantly lower than LR and LR@HA after incubation with H_2_O_2_ through detecting the fluorescent intensity of DCF by microplate reader (Fig. S10) and flow cytometric (Fig. 3a and b), revealing that the ability of PDH to scavenging ROS inside bacteria. Moreover, through ongoing monitoring the fluorescent intensity in the supernatant for 70 min, the results indicating LR@PDH possessed the capacity to remove excessive ROS in the in the environment (Fig. 3c). In addition, the ROS-scavenging ability of LR@PDH on the intracellular ROS of H_2_O_2_-stimulated HGFs cells was evaluated through a transwell system (Fig. 3d). As illustrated in Fig. 3e and f, HGFs cells treated with LR@PDH showed the lowest fluorescence intensity compare other groups, suggesting that LR@PDH could scavenge the ROS within HGFs.

### Cytoprotective effect of LR@PDH

**Fig. 4:**
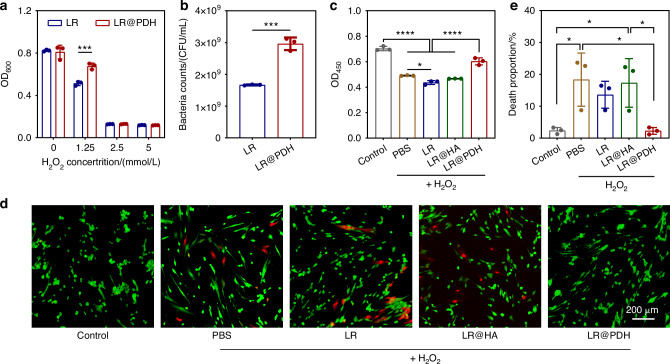
Cytoprotective ability of LR@PDH. **a** Survivals of LR and LR@PDH after exposure to different concentration of H_2_O_2_ for 12 h. **b** CFU count of LR and LR@PDH after treated with 1.25 mmol/L H_2_O_2_ for 12 h. **c** Viability of HGFs cells incubated with different treatments under H_2_O_2_ conditions after 24 h. **d** Representative images and e) quantitative analysis of live (green)/dead (red) staining of HGFs cells after treated with PBS, LR, LR@HA and LR@PDH at the presence of 200 μmol/L H_2_O_2_. Scale bar, 200 μm. *P <0.05, ***P <0.001 and ****P <0.000 1. The data are presented as mean ± SD, *n* = 3

Given that excellent biocompatibility is an essential prerequisite for the clinical application of LR@PDH in the treatment of periodontitis, the cytotoxicity of LR@PDH on human gingival fibroblasts (HGFs) was systematically assessed. Utilizing the CCK-8 assay, our results demonstrated that LR, LR@HA, and LR@PDH exhibited negligible cytotoxic effects on HGFs after co-culture for 24 h, thereby confirming their biocompatibility and potential safety for use in periodontal therapy (Fig. S11). After that, the protective effect of PDH for probiotics and cells against ROS-induced damage was explored. Briefly, LR and LR@PDH were subjected to H_2_O_2_ to investigate whether the PDH layer possessed the ability to protect LR against ROS-mediated environmental assaults, the viability of LR@PDH was significantly higher than LR (Fig. 4a). Notably, the live bacteria in the LR@PDH group were almost double that of the LR group after incubation with 1.25 mmol/L H_2_O_2_ for 12 h (Fig. 4b and Fig. S12). Moreover, when investigated whether LR, LR@HA and LR@PDH could protect HGFs cells against ROS-mediated cytotoxicity, the LR@PDH-treated HGFs cells displayed significantly higher viability than HGFs cells incubated with PBS, LR and LR@HA (Fig. 4c). Similarly, from the results of cell live/dead stains assay, LR@PDH treated groups show the lowest death rate compare to groups with treatment of PBS, LR and LR@HA (Fig. 4d and e), indicating LR@PDH was able to protect HGFs cells against ROS-induced injure due to the robust ROS-scavenging activity.

### In vitro anti-bacterial effects

**Fig. 5:**
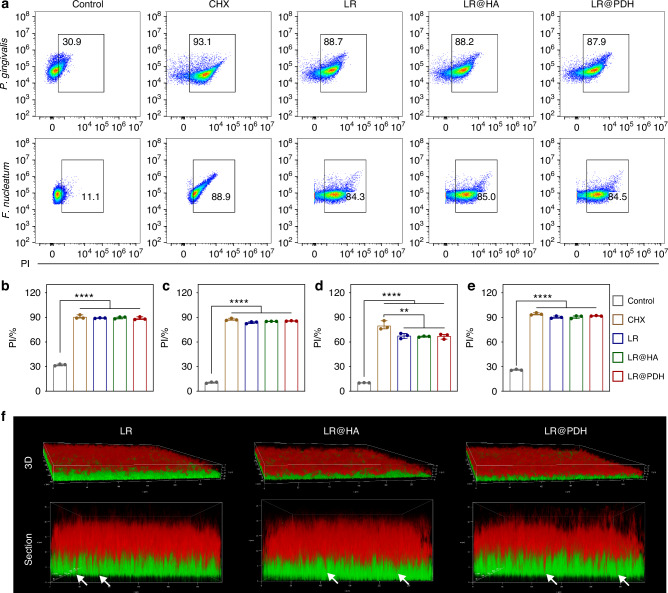
In vitro anti-bacterial effects. **a**–**e** Flow cytometry evaluated the antibacterial effects of CFS derived from LR, LR@HA, and LR@PDH via propidium iodide (PI) staining against **b** *P. gingivalis*, **c** *F. nucleatum*, **d** *S. aureus* and **e** *S. mutans*. CFS, cell-free supernatant. **f** Images of CLSM showing the penetration of LR, LR@HA, and LR@PDH (red) into biofilms (green) with treatment for 2 h. **P < 0.01 and ****P < 0.000 1. The data are presented as mean ± SD, *n* = 3

In this part, cell-free culture supernatants of LR, LR@HA, and LR@PDH was co-cultured with four pathogenic bacteria (*P. gingivalis*, *F. nucleatum*, *S. mutans* and *S. aureus*) to explore whether PDH coating affects the production of antibacterial molecule by *L. reuteri*. In order to evaluate the antibacterial ability of CFS derived from LR, LR@HA, and LR@PDH, propidium iodide (PI) staining and flow cytometry analysis were performed. As shown in the Fig. 5a–e and Fig. S13, after co-cultured with pathogenic bacteria for 8 h, the proportion of dead bacteria in LR, LR@HA, and LR@PDH groups were approximately 87.9% to 88.7% for *P. gingivalis*, 84.3% to 85.0% for *F. nucleatum*, 63.4% to 67.9% for *S. aureus*, and 89.3% to 91.7% for *S. mutans*, which demonstrated antibacterial effects comparable to chlorhexidine. Although the antibacterial effect of CFS of LR, LR@HA, and LR@PDH against *S. aureus* was inferior to chlorhexidine, they still showed a significant effect compared to the control group (MRS treatment group). Interestingly, MRS medium also showed some antibacterial effect against these four pathogenic bacteria (the proportion of dead bacteria of four pathogenic bacteria were about 9.94 to 30.9%), which possibly demonstrated that MRS medium was not suitable as a growth medium for these four pathogenic bacteria. Overall, the CFS of LR, LR@HA, and LR@PDH all demonstrated satisfactory and analogous antibacterial abilities, although subtle differences were observed. Moreover, the images observed by a confocal laser scanning microscope revealed there were significant increase of dead bacterial (labeled red) among these four pathogenic bacteria after treatment with chlorhexidine and CFS derived from LR, LR@HA, and LR@PDH (Fig. S14). Analogously, the results from spread plate assay demonstrated that the bacteria viability of four pathogenic bacteria were markedly plunged after treated with chlorhexidine and CFS derived from LR, LR@HA, and LR@PDH compare to control groups (Fig. S15). These results indicated that CFS derived from LR, LR@HA, and LR@PDH all possessed a desirable antibacterial activity against four common pathogenic bacteria, which potentially owe to the presence of reuterin and other antibacterial products in the cell-free culture supernatants, and further proved PDH coating could not lead an adverse impact on the antimicrobial property of *L. reuteri*.

### Penetration ability of LR@PDH into biofilms

In this study, the penetration ability of LR@PDH into a dual-species biofilm developed by *P. gingivalis* and *F. nucleatum* was evaluated. The LR, LR@HA and LR@PDH were stained as red by Cy5, and the bacterial biofilms were labeled as green by DMAO. After incubated with biofilms for two hours, the fluorescence images were recorded by a confocal laser scanning microscope to observe the distribution of LR, LR@HA and LR@PDH in the biofilms. As shown in Fig. 5f, the dual-species biofilm exhibited a thickness of approximately 15 μm. Cross-sectional imaging revealed a tooth-like interface between the red and green fluorescence signals, with tentacle-like red fluorescent extensions penetrating into the deeper layers of the green biofilm. These findings indicate that LR, LR@HA, and LR@PDH were capable of partially penetrating the biofilm matrix and exerting antibacterial effects within its deeper regions.

### The mucoadhesive capability of LR@PDH

**Fig. 6:**
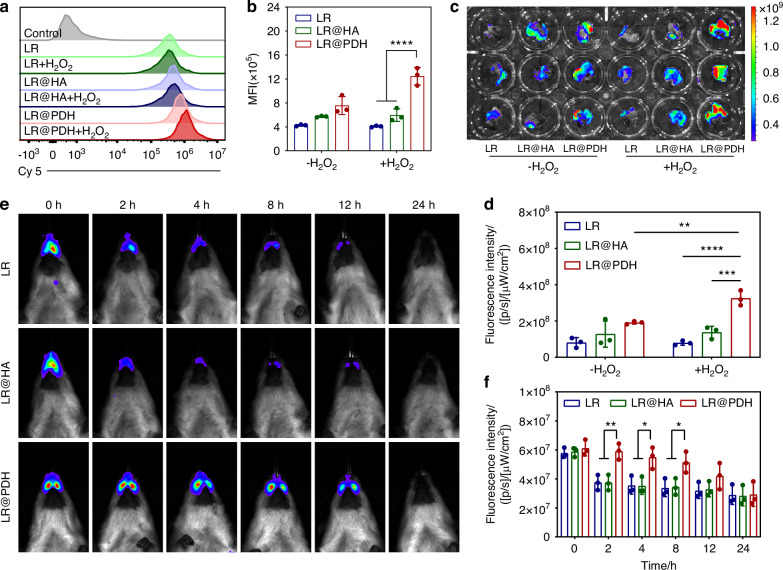
Mucoadhesive capability of LR@PDH. **a** Representative flow cytometry histograms and **b** quantification analysis of the fluorescence-positive cells after treated with Cy5-labeled LR, LR@HA and LR@PDH with or without H_2_O_2_ condition. **c** Fluorescence images and **d** quantification analysis of oral tissues after incubation with Cy5-labeled LR, LR@HA, and LR@PDH with or without H_2_O_2_ condition ex vivo. **e** Fluorescence images and **f** quantification analysis of experimental periodontitis rats after treatment with DIR-labeled LR, LR@HA, and LR@PDH for different time point. *P < 0.05, **P < 0.01, ***P < 0.001 and ****P < 0.000 1. The data are presented as mean ± SD, *n* = 3

To verify the adhesion ability of LR@PDH to gingival fibroblasts, Cy5-labeled LR, LR@HA, and LR@PDH were respectively co- cultured with HGFs cells for 4 h under the presence of H_2_O_2_ or not. Fluorescence-positive cells were analyzed using flow cytometry, and the fluorescence intensity in the LR@PDH with H_2_O_2_ group was markedly higher than in the other groups (Fig. 6a and b). In addition, freshly collected rat oral mucosal tissues were incubated with Cy5–labeled LR, LR@HA, or LR@PDH in PBS for 1 h to evaluate the adhesive ability ex vivo. As shown in Fig. 6c and d, there was no significant difference in fluorescence intensity between LR@HA and LR@PDH groups without H_2_O_2_ treatment, however, after incubation with H_2_O_2,_ the fluorescence intensity of the LR@PDH group was much higher than of the LR and LR@HA groups (Fig. 6c and d). Intriguingly, LR@PDH exhibits stronger adhesion to both cells and tissues in all treatment groups, the Cy5-labeled LR@PDH group demonstrated the most pronounced fluorescence signal, whether incubated with H_2_O_2_ or not. This potential explanation lies in the fact that the polymer PDH’s molecular structure encompasses numerous hydrophobic alkyl and aromatic groups. These groups could enhance the binding of LR@PDH to cell surfaces via hydrophobic interactions^34–36^. Furthermore, coating PDH on the surface of LR changed the distribution of surface charge, which perhaps further enhanced hydrophobic interactions^37,38^. Meanwhile, the morphology of LR, LR@HA, and LR@PDH adhered on the surface of oral mucosal tissues was observed by SEM (Fig. S16). The images captured demonstrated that the LR@PDH group showed the highest number of bacteria on the tissue surface under H_2_O_2_ treatment, further supporting the adhesive capability of LR@PDH in an inflammation state.

Due to the excellent adhesive ability of the PDH layer observed ex vivo, the intraoral retention period of LR@PDH was further explored in a periodontitis rat model. Given the high level of ROS at the periodontal inflammatory site, the exposed catechol hydroxyl group could significantly prolong the residence time of *L. reuteri*. As shown in Fig. 6e and f, due to the strong mucosal adhesive ability of the PDH coating, the fluorescence intensity of the LR@PDH group was noticeably higher than of the LR and LR@HA groups for up to 8 h, and during the 8-hour period, the fluorescence signal of the LR@PDH group faded more slowly. Additionally, the rats were euthanized after 24 h, and the intestines along with other major organs (i.e., heart, liver, spleen, lung and kidney) were collected for ex vivo IVIS imaging. As shown in Fig. S17, no fluorescence signal was detected in any major organs except for the gastrointestinal tract, indicating that LR@PDH is confined to the digestive system and had no significant adverse effects on major organs of rats.

### Therapeutic efficacy of LR@PDH against periodontitis

**Fig. 7:**
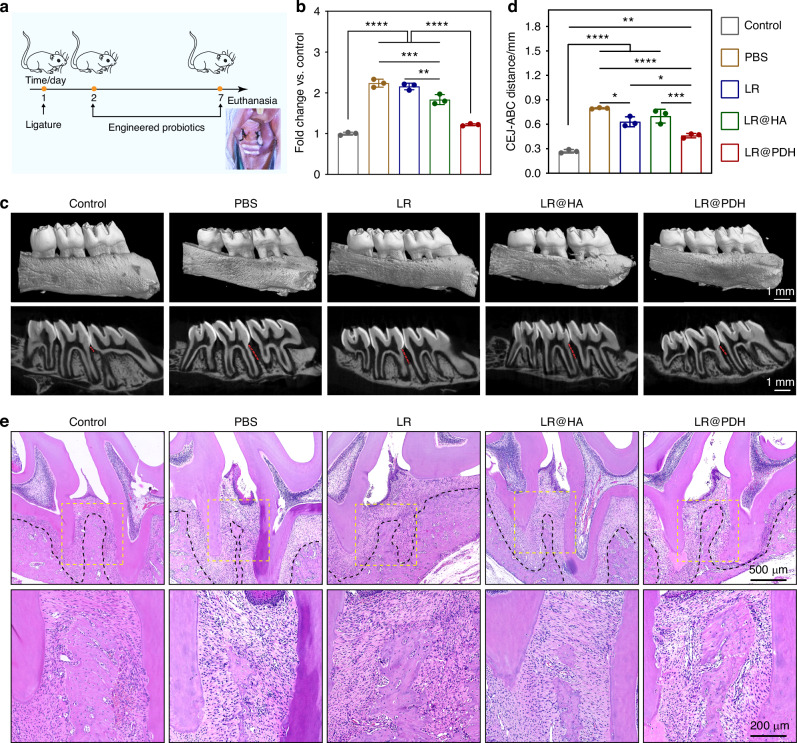
Therapeutic efficacy of LR@PDH against periodontitis. **a** Schematic of the experimental process in rat periodontitis models. **b** Changes of ROS levels in gingival crevicular fluid of rats after different treatment. **c** Micro-computed tomography (Mcro-CT) showed the rat maxillary via 3D reconstruction and two-dimension plane photography. Scale bar, 1 mm. **d** Quantitative analyses of the cementoenamel junction–alveolar bone crest (CEJ–ABC) distance. **e** HE staining of paraffin sections of periodontal tissues around ligation area. *P < 0.05, **P < 0.01, ***P < 0.001 and ****P < 0.000 1. The data are presented as mean ± SD, *n* = 3

Subsequently, the therapeutic efficacy of LR@PDH against periodontitis in vivo was assessed. The establishment of rat periodontitis models and treatment procedures was illustrated in Fig. 7a. The maxillary the first and second maxillary molars (M1, M2) was ligated by sutures and then suspensions of *P. gingivalis* was injected into the ligation sites. The ligations sites of rats were respectively treated with different formulations [PBS, LR (~10^8^ CFU/mL), LR@HA (HA: 2 mg/mL, LR: ~10^8^ CFU/mL), and LR@PDH (PDH: 2 mg/mL, LR: ~10^8 ^CFU/mL)] twice every day for a week. The rats received PBS treatment were set as the positive control, while those rats without ligation treatment served as the negative control.

As depicted in Fig. S18, the GBI scores in the LR@PDH group were significantly lower compare with PBS, LR and LR@HA-treated group though showed higher scores than healthy group, suggesting that the gingival inflammation of rats could be effectively relieved by LR@PDH. Furthermore, the periodontal relative ROS level in each group were examined by collected GCF of rats and DCFH-DA fluorescence probe was employed. Results from Fig. 7b revealed rats with treatment of LR@PDH had lower ROS levels with the lowest fluorescence intensity than other test groups, which further testified the anti-inflammatory ability of LR@PDH against periodontal inflammation in rats. Besides, a micro-computed tomography (micro-CT) was employed to scan the harvested maxillae to further assess the level of alveolar bone resorption around the first and second molars. CEJ-ABC (the vertical distance between the cementoenamel junction and the alveolar bone crest) was measured to evaluate the extent of alveolar bone resorption. As illustrated in Fig. 7c and d, PBS-treated periodontitis rats exhibited severe bone resorption compare to healthy rats, while the level of bone resorption alveolar was alleviated with LR and LR@HA treatment, which were probably given the credit to the intrinsic regulation of microbiota effects of probiotics. The alveolar bone loss in the LR@PDH-treated group was significantly alleviated compared to the PBS, LR and LR@HA treated group, which was attributed to the superior ROS-scavenging capacity and adhesive capability of LR@PDH. Benefiting from the enhanced adhesive ability, LR@PDH can act in continuously regulating periodontal dysbiosis and maintaining periodontal microbial homeostasis.

**Fig. 8:**
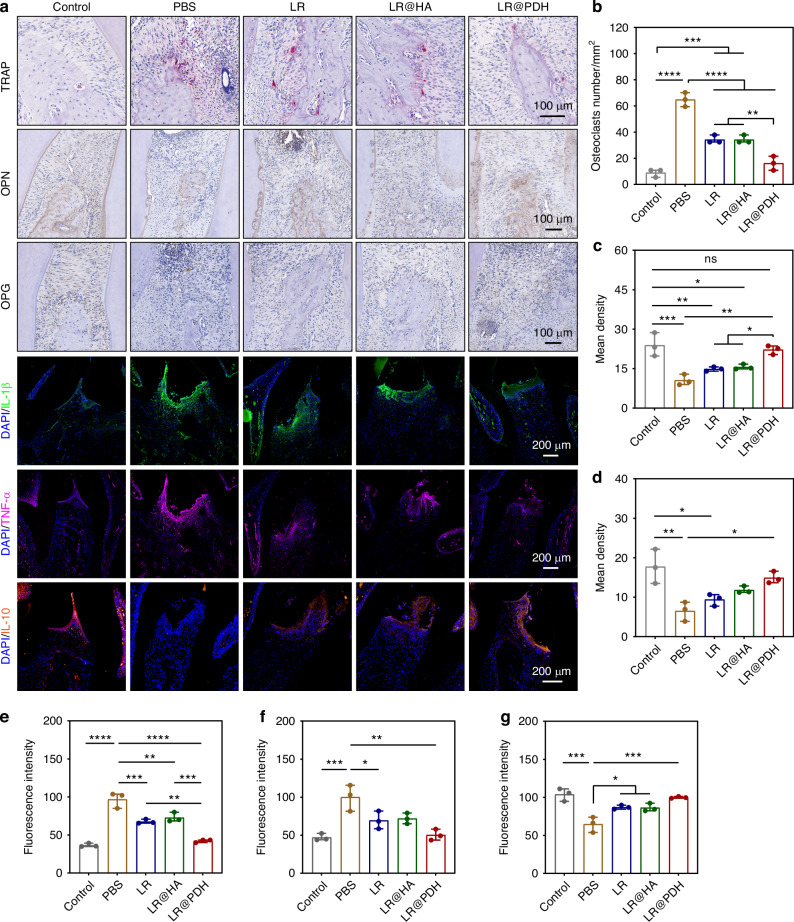
Histological evaluation of periodontal tissues around the first and second molars. **a** TRAP staining of osteoclast-like cells, IHC staining of expression of OPN and IF staining of expression of IL-1β, TNF-α and IL-10. Quantitative results of **b** TRAP-positive cells, **c** OPN levels, **d** OPG levels, **e** IL-1β levels, **f** TNF-α levels and **g** IL-10 levels. *P < 0.05, **P < 0.01, ***P < 0.001 and ****P < 0.000 1. The data are presented as mean ± SD, *n* = 3

Histological evaluation was further performed to assess the therapeutic efficacy of LR@PDH in periodontitis rats. As illustrated in Fig. 7e, the PBS-treated periodontitis group exhibited abundant inflammatory cells infiltration in the junctional epithelium, and the alveolar bone was severely destroyed, with a narrow alveolar bone width. Although alveolar bone resorption was present in the LR and LR@HA groups, they showed better alveolar ridge widths compared to the PBS group. Notably, alveolar bone resorption was prevented in rats treated with LR@PDH, resulting in significant recovery of alveolar ridge widths and heights. TRAP staining revealed a significant reduction in the number of osteoclast-like cells following treatment with probiotics. Specifically, the LR@PDH group exhibited a lower number of osteoclast-like cells compared to the LR and LR@HA groups (Fig. 8a, b). Furthermore, immunohistochemical (IHC) staining demonstrated that the levels of two key osteogenic differentiation-related factors, osteopontin (OPN) and osteoprotegerin (OPG), were significantly upregulated in the probiotics-treated groups, particularly in the LR@PDH group compared to PBS group (Fig. 8a, c and d). This finding suggests that the engineered *L. reuteri* strategy can enhance osteogenic activity during alveolar bone reconstruction. Additionally, as illustrated in Fig. 8a, e and f, the expression levels of pro-inflammatory cytokines, including IL-1β and TNF-α, were significantly reduced in the LR@PDH-treated group compared to other treatment groups. Conversely, the expression of the anti-inflammatory cytokine IL-10 was significantly increased in the LR@PDH-treated group (Fig. 8a, g).

### Modulatory effect of LR@PDH on the subgingival microbiome

**Fig. 9:**
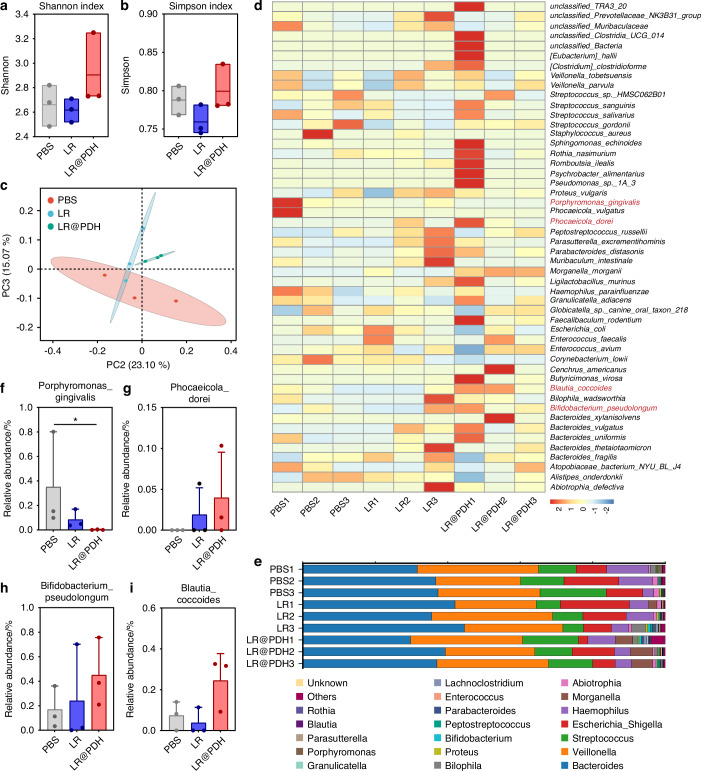
Evaluation the ability of LR@PDH to regulate subgingival flora. The subgingival microbiome α-diversity analysis through **a** Shannon index and **b** Simpson index. **c** Principal coordinates analysis (PCoA) plot (based on chord metric) to visualize the β-diversity of the subgingival microbiome in different treatment groups. Relative abundance of subgingival microbiome at **d** species and **e** genus levels in rats after different treatments. Relative abundance of **f** *P. gingivalis*, **g** *Phocaeicola_dorei*, **h** *Bifidobacterium_pseudolongum* and **i** *Blautia_coccoides* in the subgingival microbiome of rats treated with different groups. *P < 0.05. The data are presented as mean ± SD, *n* = 3

Dysregulated subgingival microflora often became the initiation factor in the development of periodontitis^39^. Therefore, due to the capacity of regulating the subgingival flora of probiotics, the changes in the subgingival microbiome composition of periodontitis rats following different treatments were further investigated. Periodontitis rats were treated with PBS, LR, and LR@PDH for 7 days. The 16S ribosomal DNA (rDNA) gene sequencing technique was applied using samples collection from silk sutures. Although no statistically significance, an increase was observed in the mean values of the subgingival microbiome α-diversity Simpson and Shannon index in the LR@PDH group comparted to the PBS- and LR-treated group, suggesting the positive effects of LR@PDH on subgingival microbiota modulation (Fig. 9a, b). Moreover, through principal coordinates (PCoA) analysis with chord metric, the β-diversity of the subgingival microbiome of rats treated with LR@PDH exhibited distinct subgingival microbiota profiles compared with other treatment groups (Fig. 9c). Fig 9d and e showed the relative abundance of subgingival microbiome among rats with different treatment at the species and genus levels, revealing LR@PDH treatment significantly decreased the relative abundance of *P. gingivalis* (known as the main pathogenic bacterium to periodontitis) (Fig. 9f). In addition, a slight increase in the average abundance of the probiotics *Blautia_coccoides* (identified as improving metabolic disorders)^40^, *Bifidobacterium_pseudolongum* (known as effectively ameliorated damage of the epithelial barrier)^41,42^, *Phocaeicola_dorei* (has function of alleviating inflammation and regulating cytokines levels)^43^ and *Lactobacillaceae* (has positive impacts on gut health by regulating the immune system and offering defense against harmful pathogens)^44^ were observed in the LR@PDH treatment group (Fig. 9g–i, Fig. S19).

### In vivo safety evaluation

The biosafety of probiotics was assessed in vivo through histological assessment for major organs (including heart, liver, spleen, lung, and kidney), the changes of body weight, and hematology analyses. As shown in Fig. S20, H&E staining of tissue sections from major organs revealed no histologically discernible inflammation or necrosis in either the treatment or control groups. Moreover, body weight curve plotted throughout the experimental period showed no significant abnormal changes in body weight among all groups (Fig. S21). Furthermore, hematology [including count of white blood cells (WBC) and red blood cells (RBC), lymphocytes (Lymph), intermediate cells (Mid), neutrophile granulocyte (Gran)] and Serological analyses [including total proteins (TP), albumin (ALB), globulin (GLO), total bilirubin (TBIL), aspartate aminotransferase (AST), alanine aminotransferase (ALT), creatinine (CRE), urea and glucose] were further performed, and the results indicated that there were no significant adverse effects after probiotics treatment compared to the health group (Figs. S22–S23).

## Discussion

This study developed an inflammation-responsive polymer-engineered *L. reuteri* system (LR@PDH) to address the elevated levels of ROS and microbial dysbiosis in the subgingival microenvironment of periodontitis. This system was designed to enhance the colonization of probiotics under oxidative stress conditions and mitigate the detrimental effects associated with periodontitis. Clinical research has shown that patients with periodontitis have significantly higher levels of ROS metabolites and total oxidant status (TOS) in serum, saliva, and GCF compared to controls^45^. Bai et al. found the levels of ROS in periodontal tissue of ligature-induced periodontitis rat was significant higher than health rat^46^. The overproduction of ROS and the imbalance of the body’s antioxidant defense system are the primary culprits of cytotoxic effects and exacerbated periodontal tissue destruction^6^.

Our in vitro experiments showed that LR@PDH exhibited strong ROS-scavenging activity due to the phenylborate ester groups within PDH. Under 400 μM H₂O₂, LR@PDH markedly reduced both intracellular and extracellular ROS levels; similarly, co-culture experiments confirmed that LR@PDH effectively decreased H₂O₂-induced ROS accumulation in HGFs. This ROS-neutralizing capacity also protected LR@PDH from oxidative damage, as LR@PDH maintained significantly higher viability than native LR in 1.25 mM H₂O₂. Consistently, CCK-8 and live/dead assays demonstrated that LR@PDH protected HGFs against ROS-induced cytotoxicity.

Previous studies have shown that cell-free supernatants (CFS) from *L. reuteri* exhibit antibacterial activity against oral pathogens, largely attributed to the broad-spectrum antimicrobial metabolite reuterin produced under anaerobic conditions^47–52^. Our findings revealed that CFS derived from LR, LR@HA, and LR@PDH displayed strong antibacterial effects against four major periodontal pathogens, indicating that polymer coating did not impair LR’s inherent antimicrobial properties. Because oral pathogens prefer to form extracellular polymeric substances (EPS)-rich biofilms that hinder drug penetration, antimicrobial agents capable of reaching deeper biofilm layers are particularly valuable^53,54^. Using a dual-species biofilm model, we found that LR, LR@HA, and LR@PDH all penetrated deep into biofilm structures within 2 h, demonstrating that LR@PDH retains the capacity to act within biofilm interiors.

In inflammatory environments, the phenyl borate groups on LR@PDH are cleaved, exposing adhesive catechol moieties that prolong bacterial retention at diseased sites. Adhesion experiments confirmed that LR@PDH exhibited significantly stronger attachment to HGFs and excised rat oral mucosa in the presence of H₂O₂, surpassing both LR and LR@HA. Moreover, LR@PDH persisted longer in the oral cavity of periodontitis rats, suggesting extended therapeutic action at inflamed sites. The enhanced adhesion arises from the catechol group’s dual non-covalent and covalent interactions with biological tissues, including reactions with nucleophilic amino and thiol groups^55,56^.

In vivo, LR@PDH demonstrated robust anti-inflammatory activity and promoted alveolar bone remodeling, suggesting an improved therapeutic effect during periodontal regeneration. This aligns with recent findings that *L. reuteri* exerts anti-inflammatory effects by suppressing TLR4-mediated cytokine production via peptidoglycan components, and enhances osteogenesis by delivering extracellular vesicles (EVs) to mesenchymal stem cells^32,57^. Reuterin has also been shown to restore the osteogenic potential of inflamed PDLSCs by modulating Cx43-related ER stress^58^. Together, these mechanisms highlight the multifaceted capacity of *L. reuteri* to regulate both inflammation and bone remodeling. In our system, the PDH coating enables ROS-responsive adhesion, allowing prolonged retention of LR@PDH at inflamed periodontal sites, where sustained release of reuterin and bioactive EVs may contribute to reducing bone resorption and enhancing periodontal regeneration^57,58^. Additionally, LR@PDH significantly modulated the subgingival microbiota composition in ligature-induced periodontitis rats, characterized by an increased abundance of beneficial taxa and a reduction in pathogenic bacteria. Given that subgingival microbial profiles vary among periodontal sites and correlate with localized inflammation severity, these microbiota-modulating effects further support LR@PDH as a promising therapeutic agent^59,60^.

Overall, LR@PDH possessed the ability to neutralize oxidative stress, mitigate periodontal inflammation, and reduce alveolar bone resorption. This inflammation-responsive probiotic strategy showed promising potential for treating other inflammatory diseases driven by bacterial infections, such as peri-implantitis and osteomyelitis, highlighting its broad applicability in the field of inflammatory disease management. Despite encouraging outcomes, several challenges remain for clinical translation. The preservation of bacterial viability during storage and transportation, as well as the optimization of clinical delivery forms (locally deliverable liquid or injectable preparation), requires further investigation. Furthermore, because our engineered probiotic system relies on a ROS-responsive polymer coating, the therapeutic efficacy may be site-dependent. Regions with higher ROS levels may experience more rapid polymer degradation, stronger adhesion, and more pronounced therapeutic benefits. Addressing these issues will be crucial to advancing this ROS-responsive LR@PDH toward practical periodontal therapy.

## Materials and methods

### Materials and reagents

Dopamine hydrochloride (DA), N-Hydroxysuccinimide (NHS), phenylboric acid (PA), hyaluronic acid (HA) and 1-(3-Dimethylaminopropyl)-3-ethyl carbodiimide hydrochloride (EDC) were sourced from Sigma-Aldrich (St. Louis, USA). PrestoBlue™ Cell Viability Reagent was purchased from ThermoFisher Scientific. 2,7-Dichlorofluorescin diacetate (DCFH-DA), Cell Counting Kit-8 (CCK-8), N, N-dimethylaniline N-oxide (DMAO), Propidium Iodide (PI) and Calcein Acetoxymethyl Ester (Calcein AM) was obtained from Beyotime Biotechnology (Shanghai, China). 4-(4,4,5,5- tetramethyl-1,3,2 dioxaborolan-2-yl) benzyl bromide and Di-tert butyl dicarbonate [(Boc)_2_O] were obtained from TCI (Tokyo, Japan). Poly (allylamine hydrochloride) and chlorhexidine acetate was sourced from Aladdin (shanghai, China). 4-Trifluoroacetic acid (TFA) was purchased from Alfa Aesar (Ward Hill, USA). MRS broth was obtained from Huangkai Microbial (Guangdong, China), BHI broth was sourced from Beijing Land Bridge Technology (Beijing, China), Tryptic Soy Broth (Soybean-Casein Digest Medium) was obtained from BD Bacto (USA), Tryptic Soy Agar, hemin, vitamin K1, NHS-Cyanine 5 (Cy-5), Hoechst 33342 and defibrinated sheep blood were purchased from Solarbio (Beijing, China). L-cysteine was sourced from Biofroxx (Beijing, China), yeast extract was obtained from Oxoid (England).

### The ROS detection in health and periodontitis rat

The detailed process of constructing the rat periodontitis model is described in Section “Animal Model”. Briefly, sterile cotton swabs were used to collect GCF from healthy and periodontitis rats. After being placed in periodontal pocket around the first and the second molar for 5 s, the swabs were transferred to 1.5 mL centrifuge tubes containing 200 µL of sterile phosphate buffered saline (PBS). The tubes were then centrifuged at 5000 rpm for 5 min, and the supernatant was collected. A fluorescence probe, DCFH-DA (10 mmol/L), was used to detect the levels of ROS in the saliva samples from different groups, with fluorescence signals monitored using a microplate reader.

### Bacteria culture

*Lactobacillus reuteri* (*L. reuteri*), *Porphyromonas gingivalis* (*P. gingivalis*), *Fusobacterium nucleatum* (*F. nucleatum*), *Streptococcus mutans* (*S. mutans*) and *Staphylococcus aureus* (*S. aureus*) were provided by the School of Stomatology, Wuhan University (China). *L. reuteri* was cultured in liquid MRS medium and MRS agar plates at 37 °C. *P. gingivalis* was cultured in Tryptic Soy Broth (TSB) as liquid medium and Tryptic Soy Broth (TSB) agar medium supplemented with 5% defibrinated sheep blood as solid medium. Both the liquid and solid medium were supplemented with, L-cysteine (0.5 g/L), yeast extract (5 g/ L), hemin (5 mg/L) and vitamin K1 (1 mg/L). *F. nucleatum* was grew in the liquid Brain Heart Infusion (BHI) broth and Tryptose Soya Agar supplemented with 5% sheep blood solid medium. *P. gingivalis* and *F. nucleatum* were both grew at 37 °C with anaerobic condition. *S. mutans* and *S. aureus* were both cultured in the BHI broth or BHI agar plates at 37 °C.

### Cell culture

Human gingival fibroblasts (HGFs) were extracted according to our previous research^61^. HGFs were cultured in Dulbecco’s modified Eagle medium (DMEM, Gibco) supplemented with 1% penicillin-streptomycin (Gibco) and 10% fetal bovine serum (FBS, Gibco) in humidified atmosphere with 5% CO_2_ at 37 °C.

### Preparation and characterization of LR@PDH

The preparation of phenylboric acid-dopamine-hyaluronic acid (PDH) were described in Supporting Information. Single colonies of *L. reuteri* (LR) was picked and cultured in MRS liquid medium for 24 h. In order to conjugate the PDH onto the surface of LR, poly (allylamine hydrochloride) (PAH) was employed as a connector. Firstly, following three washes with PBS buffer solution (5 000 r/min, 5 minutes), LR was respectively incubated with different PAH concentration (0.125 and 0.25 mg/mL) for 10 min at 37 °C. Spread plate method was applied to explore the appropriate concentration of PAH to construct LR@PAH. Then, the bacterial solution was washed three times with PBS buffer solution (5 000 r/min, 5 min) to remove surplus PAH. Subsequently, the LR solution was incubated with different concentration of PDH (0.25, 0.5, 1 and 2 mg/mL) for 2 h with 250 r/min. The appropriate concentration of PDH to prepare LR@PDH was confirmed by spread plate method. After three additional washes with PBS buffer solution (5 000 r/min, 5 min) to remove superfluous PDH, and LR@PDH was obtained.

The size and electric potential of LR and LR@PDH were recorded by the Malvern Zetasizer (Nano-ZSP, British). The morphology structures of LR@PDH were observed through Field Emission Scanning Electron Microscope (SEM, Zeiss SIGMA, British) and Transmission Electron Microscope (TEM, JEM-2100, Japan). The UV-3700 UV–vis spectrophotometer (PerkinElmer, Lambda Bio 40) was employed to confirm the presence of HA and PDH on LR. After treating LR@PDH (PDH: 2 mg/mL, LR: ~10^8^ CFU/mL) with 400 μmol/L H_2_O_2_ for 30 min, the supernatant was detected by inductively coupled plasma mass spectrometry (ICP-MS, Analytik Jena, Germany) to perform analysis of boron (B) element. The element distributions of B, C, N, and O were observed by energy dispersive spectrometer mapping (JED-2300T, JEM-F200, Japan). Furthermore, LR@PDH constructed by Cy5-labeled LR ( ~ 10^8^ CFU/mL) and FITC-labeled PDH (PDH: 2 mg/mL) was observed by confocal laser scanning microscope (CLSM) (FV1200, Olympus Corporation, Japan), and FITC-labeled PDH (PDH: 2 mg/mL) coated on the surface of LR (~10^8^ CFU/mL) was analyzed by flow cytometer (Cytoflex, Beckman, USA).

### Assessment of ROS scavenging ability of LR@PDH in vitro

Firstly, the growth curves of LR, LR@HA and LR@PDH were drew to assess the effect of PDH on LR growth and proliferation. Briefly, 100 μL bacterial solution of LR (~10^8^ CFU/mL), LR@HA (HA: 2 mg/mL, LR: ~10^8^ CFU/mL) and LR@PDH (PDH: 2 mg/mL, LR: ~10^8^ CFU/mL) were inoculated in fresh MRS medium, and the bacterium were incubated at 37 °C for 12 h. The OD values of the culture medium were recorded by using a microplate reader at 600 nm every 1-h intervals.

Then, the lifetime of LR, LR@HA, and LR@PDH was investigated using PrestoBlue (Thermo Fisher Scientific), a cell viability reagent that evaluates the metabolic activity of living cells. In brief, LR (~10⁸ CFU/mL), LR@HA (HA: 2 mg/mL, LR: ~10⁸ CFU/mL), and LR@PDH (PDH: 2 mg/mL, LR: ~10⁸ CFU/mL) were inoculated in sterile PBS at 37 °C for 7 days, and the metabolic activity of LR and modified LR was monitored at 12-h intervals. Specifically, 90 μL of bacterial suspension was incubated with 10 μL of PrestoBlue reagent at 37 °C for 30 min, and the fluorescence intensity was recorded using a microplate reader (excitation: 560 nm; emission: 590 nm).

ROS scavenging ability of LR@PDH on LR: In brief, after incubating in PBS containing 400 μM H_2_O_2_ at 37 °C for 30 min, suspensions of LR (~10^8^ CFU/mL), LR@HA (HA: 2 mg/mL, LR: ~10^8^ CFU/mL) and LR@PDH (PDH: 2 mg/mL, LR: ~10^8^ CFU/mL) were centrifugated at  5 000 r/min for 3 min, after washing with PBS for three times, the sediment of bacterium were resuspended in PBS containing DCFH-DA (10 μmol/L) at 37 °C in an incubator for 30 minutes. The fluorescent signals were detected by microplate reader and Flow cytometer (Cytoflex, Beckman, USA). Moreover, the ROS levels in outside environment was assessed after treatment with LR@PDH. Briefly, LR (~10^8^ CFU/mL), LR@HA (HA: 2 mg/mL, LR: ~10^8^ CFU/mL) and LR@PDH (PDH: 2 mg/mL, LR: ~10^8^ CFU/mL) were incubated in PBS containing 400 μmol/L H_2_O_2_ at 37 °C for 30 minutes, then, bacterial suspensions were centrifugated at 5 000 r/min for 3 min and the supernatants were filtrated by sterile filters (pore size 0.22 μm). The ROS levels were detected by incubating supernatants with DCFH-DA fluorescent probe (10 μmol/L) at 37 °C for 30 min. A microplate reader (BioTek Synergy H1, excitation: 490 nm and emission: 520 nm) was applied to monitor the fluorescent signals for 70 min.

ROS scavenging ability of LR@PDH on cells: to evaluate the ability of LR@PDH of scavenging intracellular ROS, HGFs cells were seeded in 24-well plates and incubated with the following additions under 200 μmol/L H_2_O_2_ at 37 °C: (1) LR (~10^8^ CFU/mL); (2) LR@HA (HA: 2 mg/mL, LR: ~10^8^ CFU/mL); and (3) LR@PDH (PDH: 2 mg/mL, LR: ~10^8^ CFU/mL). After 24 h, DCFH-DA (10 μmol/L) was added and the cells were incubated at 37 °C for 30 min. The fluorescent images were obtained by using inverted fluorescence microscope.

### Assessment of cytoprotective ability of LR@PDH in vitro

Protective ability of LR@PDH on LR: the protective effect of PDH for LR against ROS-induced damage was evaluated. In brief, 300 μL bacterial solution of LR (~10^8^ CFU/mL) and LR@PDH (PDH: 2 mg/mL, LR: ~10^8^ CFU/mL) were inoculated in fresh MRS medium containing different concentrations of H_2_O_2_ (1.25, 2.5 and 5 mmol/L). After incubation for 12 h at 37 °C, the OD_600_ values of culture medium from different groups were recorded by a microplate reader. Furthermore, the bacterial solution was washed by PBS and then spread on MRS agar plates after sequential 10-fold dilutions.

Protective ability of LR@PDH on normal cells: By using a transwell system, the biocompatibility of LR, LR@HA, and LR@PDH to HGFs cell was evaluated through a cell counting kit-8 (CCK-8, Beyotime Biotechnology, shanghai, China). In brief, HGFs cells (5 × 10^4^ cells) were respectively co-cultured with LR, LR@HA, and LR@PDH for 24 h, and the cell activity of different groups was assessed. In addition, the ability of LR@PDH protected HGFs cells against ROS-induced damage was explored. Specifically, HGFs cells (5 × 10^4^ cells) were seeded in the 24-well plates and cultured overnight. After that, the HGFs cells were respectively co-cultured with LR, LR@HA, LR@PDH comply with the condition of 200 μmol/L H_2_O_2_ for 24 h. Then, the cell activity of different groups was assessed via CCK-8. Moreover, the cytoprotective effect of LR@PDH was also evaluated by using cell live/dead assay. Briefly, HGFs cells HGFs cells (5 × 10^4^ cells) were co-cultured with LR, LR@HA, LR@PDH in 24-well plates under the condition of 200 μmol/L H_2_O_2_ at 37 °C for 24 h. Subsequently, after removing the transwell system, the cells were washed with PBS for two times. Calcein AM (indicating live cell as green) and PI (indicating dead cell as red) were applied and the fluorescence signals were captured through an inverted fluorescence microscope (Olympus, Japan).

### Anti-pathogenic bacterial experiment

According to prior studies, *Lactobacillus reuteri*-derived cell-free supernatant (CFS) possess the properties against pathogenic bacteria attributed to the presence of reuterin^47^. Therefore, the anti-pathogenic bacterial ability of CFS from LR, LR@HA and LR@PDH were evaluated. Briefly, LR (~10^8^ CFU/mL), LR@HA (HA: 2 mg/mL, LR: ~10^8^ CFU/mL) and LR@PDH (PDH: 2 mg/mL, LR: ~10^8^ CFU/mL) were transferred to fresh MRS medium at 37 °C for 8 h, after that, the suspensions were centrifugated at 5 000 r/min for 10 min and the cell-free supernatants (CFS) were obtained by using a 0.22 μm sterile membrane filters. Furthermore, the cell-free supernatants (CFS) were spread on MRS agar plates to confirm the absence of live bacteria.

Briefly, 1 mL of *P. gingivalis* (~10^8^ CFU/mL), *F. nucleatum* (~10^8^ CFU/mL), *S. mutans* (~10^8 ^CFU/mL) and *S. aureus* (~10^8^ CFU/mL) suspension were centrifugated at 5 000 r/min for 5 mins and the bacteria cells were washed using PBS for 2 times. Later, the four pathogenic bacteria were respectively mixed with following: (1) fresh MRS medium (control group); (2) fresh MRS medium containing 0.12% chlorhexidine (CHX group); (3) LR-derived CFS (LR group); (4) LR@HA-derived CFS (LR@HA group); (5) LR@PDH-derived CFS (LR@PDH group). After 8 h, the bacteria cells were washed by PBS for three times with centrifugating at 5 000 r/min for 5 min, and 100 μL of serial decimal dilutions bacterial solution was spread on the agar plates.

Moreover, the anti-bacterial property of CFS derived from LR, LR@HA, and LR@PDH was also assessed by live/dead bacterial staining assay. DMAO (indicating live and dead bacteria cells as green) and PI (indicating dead bacteria cells as red) were employed and the fluorescence signal were captured through flow cytometric (Cytoflex, Beckman, USA) and confocal laser scanning microscope (CLSM) (FV1200, Olympus Corporation, Japan).

### Penetration into biofilms experiment

In this study, dual-species biofilms of *P. gingivalis* and *F. nucleatum* were established in vitro to assess the penetration ability of LR@PDH. Briefly, confocal dishes containing 1 mL of *F. nucleatum* (~10^7^ CFU/mL) suspension were incubated anaerobically at 37 °C for 48 h. Then, the liquid supernatant was gently removed and 1 mL of *P. gingivalis* (~10^7^ CFU/mL) was gently added in the confocal dishes. After anaerobic incubation 2 days, the dual-species biofilms were formed.

After the biofilms were developed, fluorochrome DMAO was employed to dye the dual-species biofilms as green. Subsequently, 200 μL of Cy5-labeled LR (~10^8^ CFU/mL), LR@HA (HA: 2 mg/mL, LR: ~10^8^ CFU/mL) and LR@PDH (PDH: 2 mg/mL, LR: ~10^8^ CFU/mL) were respectively slowly added in confocal dishes and incubated at 37 °C for 2 h. And then, CLSM (Leica, Germany) was used to observed the morphology of the dual-species biofilms and the distribution of LR, LR@HA, and LR@PDH.

### Assessment the adhesion in vitro

In brief, HGFs cells (~10^5^ cells) were seeded in the 6-well plates and cultured at 37 °C overnight, then, Cy5-labeled LR (~10^8^ CFU/mL), LR@HA (HA: 2 mg/mL, LR: ~10^8^ CFU/mL) and LR@PDH (PDH: 2 mg/mL, LR: ~10^8^ CFU/mL) were added in each well with incubation for 1 h at 37 °C. Then, HGFs cells of each well were collected to evaluated the adhesive condition of different groups by conducting flow cytometry.

Furthermore, the mucoadhesive ability of LR, LR@HA and LR@PDH were also explored. Specifically, about 0.5 cm^2^ of freshly separated rat mouth mucosa tissues was placed in 24-well plates after washing with PBS for three times. Subsequently, 1 mL Cy5-labeled LR (~10^8 ^CFU/mL), LR@HA (HA: 2 mg/mL, LR: ~10^8^ CFU/mL) and LR@PDH (PDH: 2 mg/mL, LR: ~10^8 ^CFU/mL) were respectively added in each well and incubation for 1 h at 37 °C, later, the mouth mucosa tissues were washed 10 min by deionized water. The fluorescence signal from different groups were captured by a small animal imaging system (PerkinElmer, IVIS Spectrum, USA). Besides, the morphology of the bacteria adhered on the rat mouth mucosa tissues were observed through a scanning electron microscope (SEM).

### Assessment of adhesion in vivo

In brief, 100 μL of DIR-labeled LR (~10^8^ CFU/mL), LR@HA (HA: 2 mg/mL, LR: ~10^8^ CFU/mL) and LR@PDH (PDH: 2 mg/mL, LR: ~10^8^ CFU/mL) were orally injected to the periodontal sock around maxillary molars of periodontitis rats to further explore the mucosal adhesion ability of LR@PDH in vivo, and the fluorescent signals were captured by IVIS to track the distribution of bacteria. Rats were randomly divided into three groups, and each rat were respectively treated with LR, LR@HA, and LR@PDH. Photos were taken at 0, 2, 4, 8, 12 and 24 h by IVIS. At the end of time, the rats were euthanized and main organs (heart, liver, spleen, lung and kidney) and gastrointestinal tract were collected for imaging.

### Animals model

The Ethics Committee of School of Stomatology, Wuhan University approved the animal experiments (Project Number: S07924070I). In brief, specific pathogen-free (SPF) female Sprague-Dawley (SD) rats with 6-week-old were purchased from Hubei Experimental Animal Research Center. Periodontitis rat model was built according to our previous study^15,61^. Briefly, the rats were anesthetized with pentobarbital sodium (30 mg/kg), 3–0 silk sutures were ligated around the cervix of the first and second maxillary molars (M1, M2) by utilizing “∞-ligation” approach, and 100 μL suspensions of *P. gingivalis* (10^8^ CFU/mL) were injected into the gingival sulcus. The silk sutures ligations were checked each day and maintained for 7 days. Rats were randomly divided in five groups as following: (1) healthy rats (control group); periodontitis rat were treated with (2) PBS, 3) LR (~10^8^ CFU/mL), (4) LR@HA (HA: 2 mg/mL, LR: ~10^8^ CFU/mL) and (5) LR@PDH (PDH: 2 mg/mL, LR: ~10^8^ CFU/mL). For all treatment group, 100 μL of PBS or bacteria suspension were injected at the ligature site of rats and repeated twice a day for 7 days. After a week treatment, the gingival inflammation of rats was evaluated by gingival bleeding index (GBI). Subsequently, all rats were euthanatized and the maxillary, blood specimens and vital organs (heart, liver, spleen, lung and kidney) were collected for further evaluation. Furthermore, the silk sutures from rats of PBS, LR and LR@PDH groups were harvested for subgingival microbial diversity analysis.

### Evaluation of ROS scavenging in vivo

At the end of treatment, the GCF of rats were collected by sterile cotton swab. In brief, the sterile cotton swab was dived into the periodontal pockets of the first and second maxillary molars and remain for 5 s, after that, the sterile cotton swab was collected in the 1.5 mL centrifuge tube with 200 μL of PBS. Then, the centrifuge tubes were centrifugated at 5 000 r/min for 5 min and obtained the supernatant. A fluorescence probe of DCFH-DA was employed to detected the levels of GCF of rats from different groups, and the fluorescence signal were monitored by a microplate reader.

### Micro CT analysis

The collected maxillary tissues were fixed in 4% paraformaldehyde for 48 h, and then, the samples were scanned by using a Micro-CT scanner (Skyscan 1276, Bruker, Germany). The scanning settings were an 80 kV operating voltage, a 45 mA current and a slice thickness of 8 μm. Furthermore, other software including DataViewer and CTvox were applied to performed two-dimensional and three-dimensional reconstruction imaging.

### Histological staining

After treatment with 4% paraformaldehyde, the maxillary samples were treated by 10% ethylene diamine tetraacetic acid (EDTA) solution for 8 weeks to decalcify. Later, gradient alcohol method was used to perform dehydration procedure and the samples were embedded in paraffin to prepare tissue slices. Hematoxylin-Eosin (H&E) staining was conducted to evaluate periodontal inflammation and tartrate-resistant acid phosphatase (TRAP) staining was performed to observe osteoclast (OC) cells around the periodontal bone tissue. Furthermore, immunofluorescent (IF) staining and immunohistochemistry (IHC) staining were conducted to assess the inflammation levels in the periodontal tissues.

### In vivo biosafety assessment

Blood samples of all rats were analyzed by blood biochemistry Celercare V5 (MNCHIP, China) and hematology analyzer (iCubio, China). Furthermore, vital organs (including heart, liver, spleen, lung, and kidney) were subjected to H&E staining to assess the biosafety in vivo.

### 16S rDNA sequencing

After 7 days, rats with different treatment were euthanatized and the silk sutures were collected for further analysis. The samples were promptly frozen in liquid nitrogen. After collect the total DNA of each sample, PCR method was utilized to amplify the microbiota V3-V4 hyper variable regions of 16S rDNA (338 F: 5’-ACTCCTACGGGAGGCAGCA-3’ and 806 R: 5’-GGACTACHVGGGTWTCTAAT-3’). After Purification, quantification, and homogenization, the sequencing library was constructed. Then, Illumina NovaSeq (Illumina NovaSeq 6000) was used to performed subsequent sequencing analyses. The 16S rDNA sequencing was based on Beijing Biomarker Technologies Co., LTD. and under the company’s standard protocols.

### Statistical analysis

GraphPad Prism (version 10.1.2) software used to perform statistical analyses. Significance among two groups was compared by using a two-tailed student’s *t* test and multiple groups comparison were calculated by one-way analysis of variance (ANOVA) test with Tukey’s multiple comparisons test. The data are presented as mean ± standard deviation (SD), and the significant difference was set as *P <0.05, **P <0.01, ***P <0.001 and ****P <0.000 1.

## Supplementary information

Supporting Information

## Data Availability

Additional data supporting the findings of this study are available from the corresponding author upon reasonable request.
