# Supplementary material for: Engineered Lactobacillus reuteri for scavenging reactive oxygen species and modulating oral microflora in periodontitis therapy
Source: Int J Oral Sci. 2026 Feb 10;18:16. doi: 10.1038/s41368-025-00418-z (PMC12887038; doi:10.1038/s41368-025-00418-z)
Supplement: Supplementary file 1 — Supporting Information [file 41368_2025_418_MOESM1_ESM.pdf]

Supporting Information

Engineered *Lactobacillus reuteri* for Scavenging Reactive Oxygen Species and Modulating Oral Microflora in Periodontitis Therapy

Yuqiang Wang [a, 1], Ying Tang [a, 1], Qianxiao Huang [b], Jiaxin An [b], Yueli Zhou [a], Hongye Yang [a], Fangfang Song [a], Xianzheng Zhang [b, *], Cui Huang [a, *]

a. State Key Laboratory of Oral & Maxillofacial Reconstruction and Regeneration & Key Laboratory of Oral Biomedicine Ministry of Education & Hubei Key Laboratory of Stomatology & School & Hospital of Stomatology, Wuhan University

b. Key Laboratory of Biomedical Polymers of Ministry of Education & Department of Chemistry, and Institute for Advanced Studies, Wuhan University

[1] These authors contributed equally to this work.

***Corresponding Author:**

Xianzheng Zhang, Key Laboratory of Biomedical Polymers of Ministry of Education & Department of Chemistry, and Institute for Advanced Studies, Wuhan University, Wuhan, Hubei, PR China. E-mail: xz-zhang@whu.edu.cn

Cui Huang, Department of Prothodontics, Hospital of Stomatology, Wuhan University, Wuhan, Hubei, PR China. E-mail: huangcui@whu.edu.cn

Experimental Section

Preparation of boc protected DA (Boc-DA)

Dopamine hydrochloride (DA, 5 mM) was dissolved in 20 mL of methanol under a nitrogen atmosphere. 10 mM triethylamine was added, and $(Boc)_2O$ (11 mM) was gradually dropped into the above solution in an ice bath environment. After the mixture was stirred at room temperature for 4 hours, methanol was removed by rotary evaporation. The obtained solid was dissolved in chloroform, extracted three times with ultrapure water, and DA(Boc) was collected after rotary evaporation.

Preparation of Boc-DA-PA

Boc-DA (5 mM) was dissolved in 15 mL of anhydrous DMF and cooled to 0 °C. K_2CO_3 (12 mM) was added to the above solution. After stirring at 0 °C for 10 minutes, 5 mL of 4 (4, 4, 5, 5-tetramethyl-1, 3, 2-dioxaborolan-2-yl) benzyl bromide (10 mM) dissolved in DMF was added. The mixture was stirred at room temperature overnight. After the reaction was completed, DMF was removed by rotary evaporation. The harvested solid was dissolved in ethyl acetate, washed three times with saturated NH_4Cl, and dried over anhydrous $MgSO_4$. The crude product was purified by silica gel chromatography, with the mobile phase of ethyl acetate/hexane = 1: 4.

Preparation of DA-PA

Boc-DA-PA (0.7 mM) was dissolved in 8 mL of DCM/TFA (1:3, vol/vol). The mixture was stirred at room temperature for 2 hours. Subsequently, 4 mL of DCM was added, and the solvent was removed by rotary evaporation.

Preparation of PA-DA-HA (PDH)

Hyaluronic acid (HA) (0.48 mM) was dissolved in DMSO, followed by the addition of EDC (2.4 mM) and stirring for 0.5 hours. Subsequently, NHS (2.4 mM) and PA-DPA (0.96 mM) were added to the above solution and stirred for 12 hours. The final product was obtained after dialysis, dehydration, and freeze-drying.

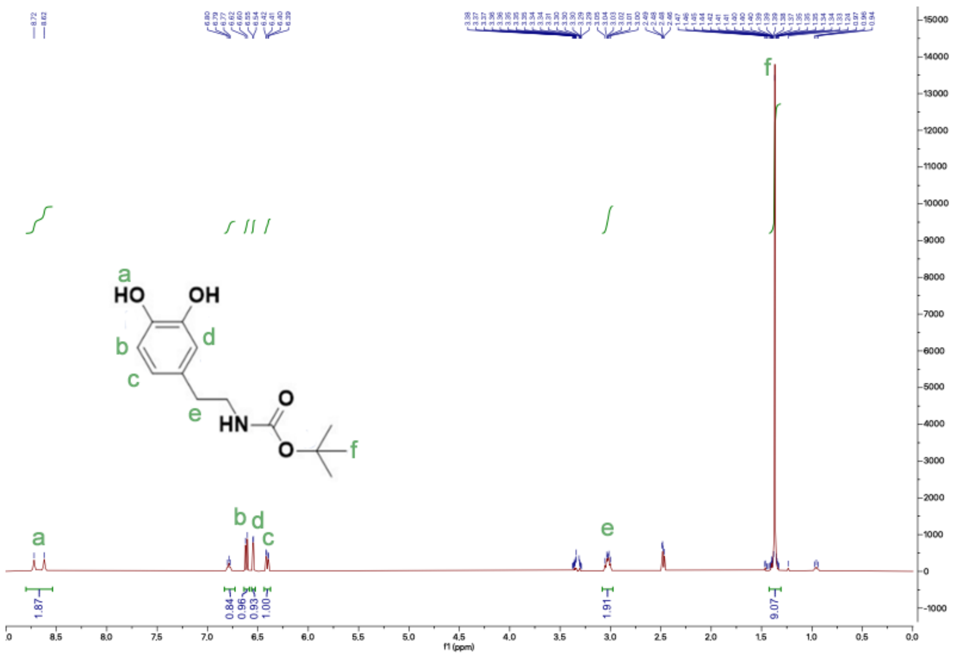

Fig. S1. ^{1}H NMR spectra of Boc-DA.

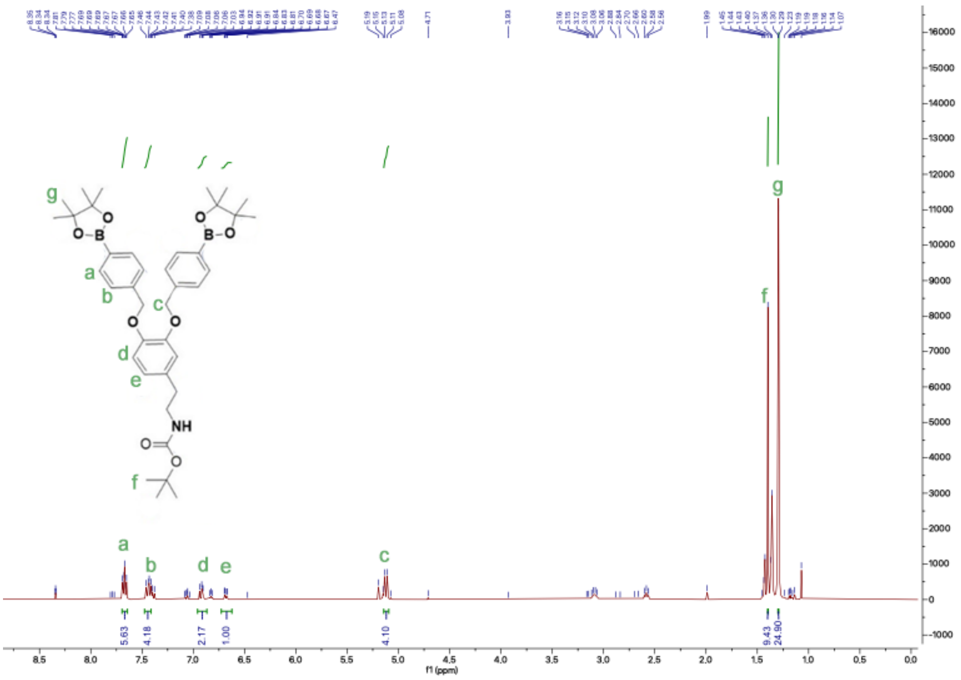

Fig. S2 . ^{1}H NMR spectra of Boc-DA-PA.

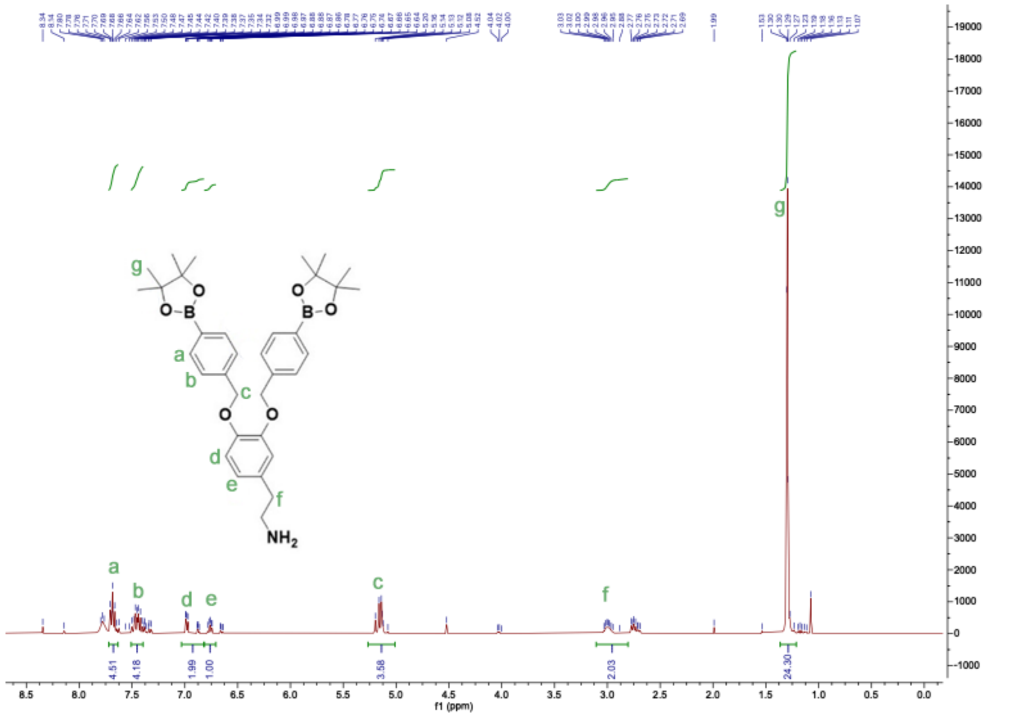

Fig. S3. ^{1}H NMR spectra of PA-DA.

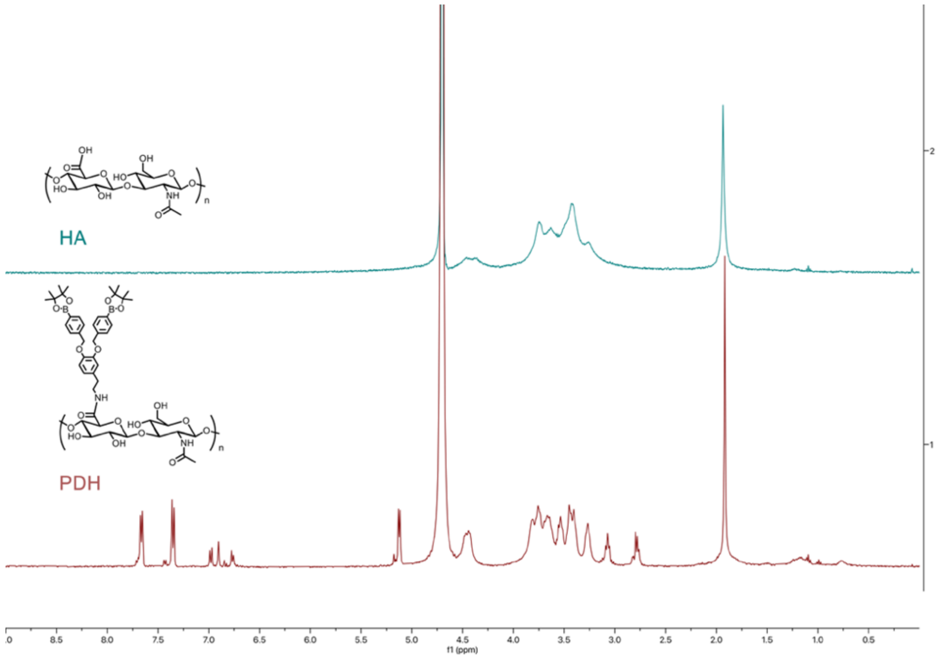

Fig. S4. ^{1}H NMR spectra of HA and PDH.

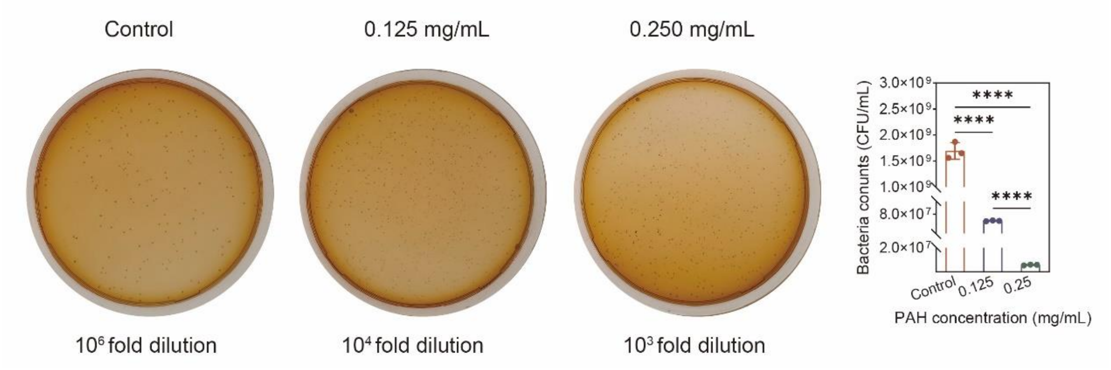

Fig. S5 Representative photographs and CFU counts of colonies of LR@PAH on MRS agar plates were taken using different concentrations of PAH by the spread plate method.

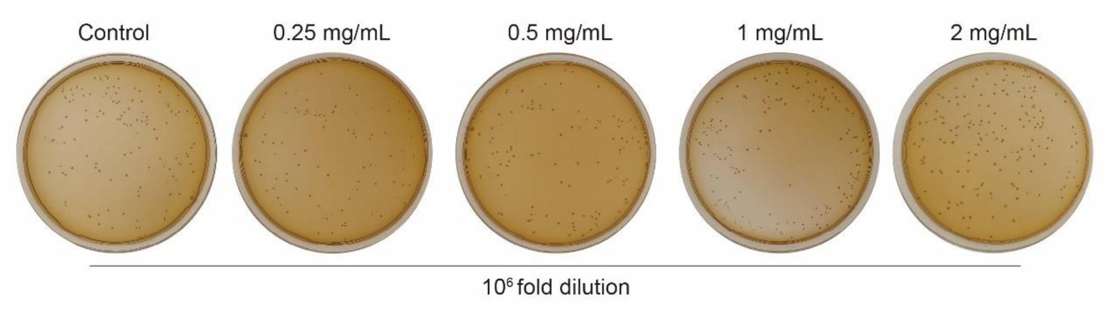

Fig. S6 Representative photographs of colonies of LR@PDH on MRS agar plates were taken using different concentrations of PDH by the spread plate method.

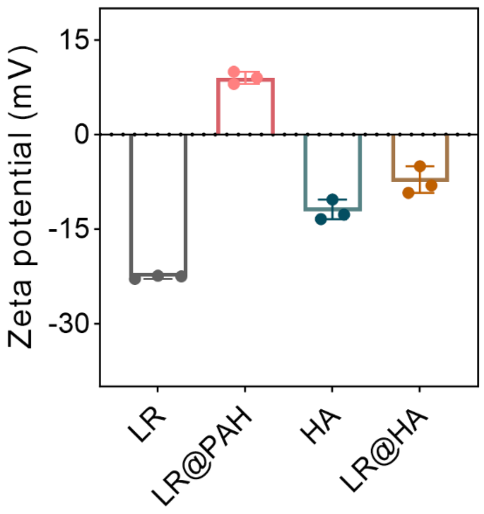

Fig. S7 Zeta potentials of LR, LR@PAH and LR@HA.

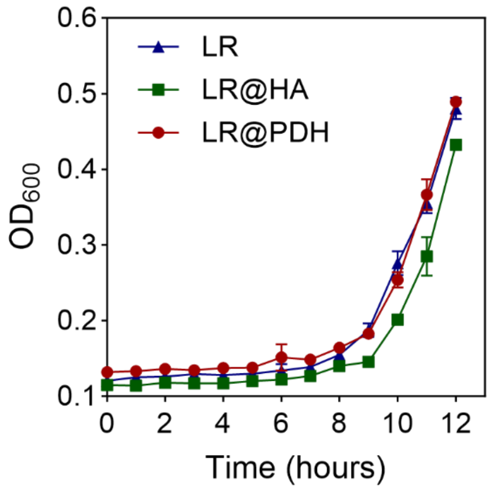

Figure S8 The growth curves of LR, LR@HA, and LR@PDH in MRS medium at 37°C. The data are presented as mean ±SD, n = 3.

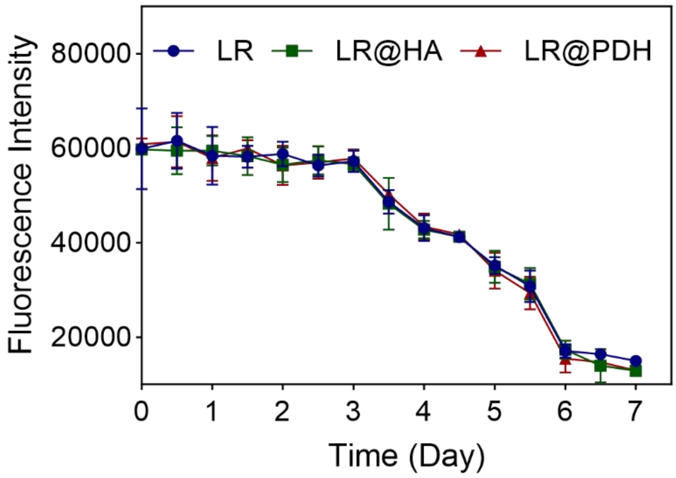

Fig. S9 The metabolic activity of LR, LR@HA, and LR@PDH. The data are presented as mean ±SD, n = 3.

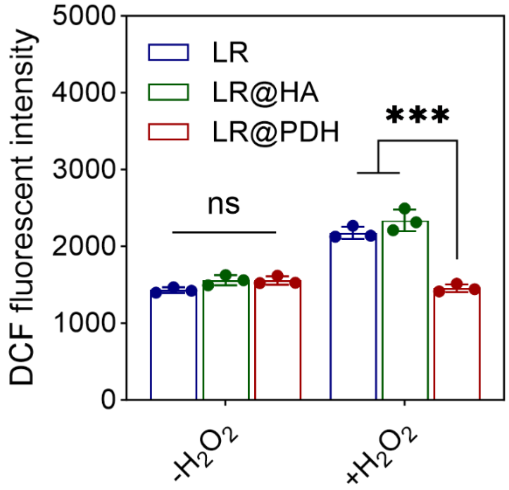

Figure S10 DCF fluorescence intensity within LR, LR@HA and LR@PDH after incubation with H_2O_2 were detected by microplate reader. ***$p < 0.001$. The data are presented as mean ±SD, n = 3.

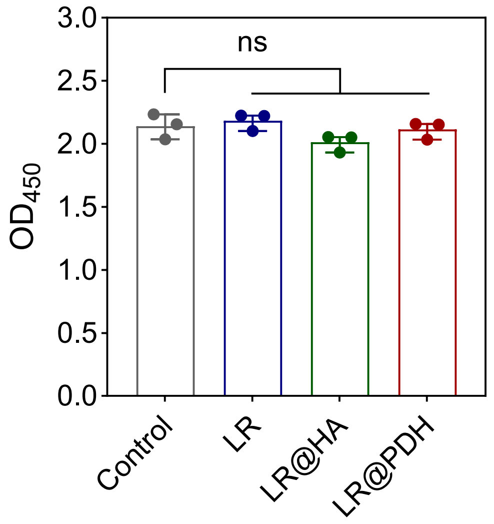

Fig. S11. Viability of HGFs cells after co-culture with LR, LR@HA, and LR@PDH to for 24 hours. The data are presented as mean ±SD, n = 3.

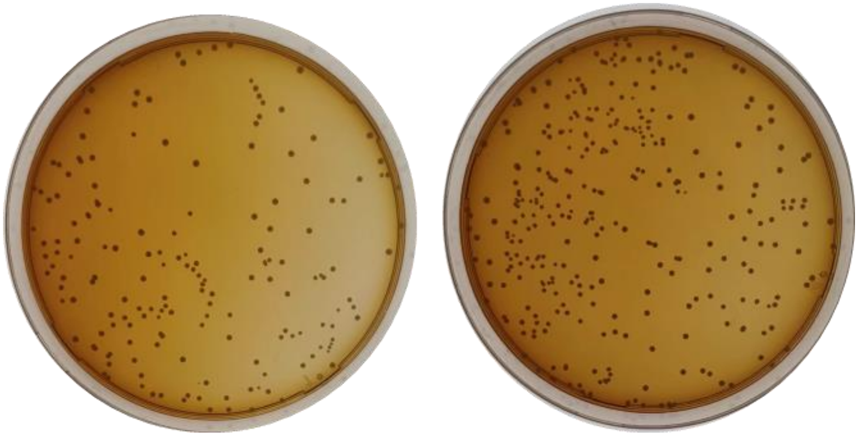

Figure S12 Representative photos of colonies of LR and LR@PDH on MRS agar plates diluted 10^6 fold.

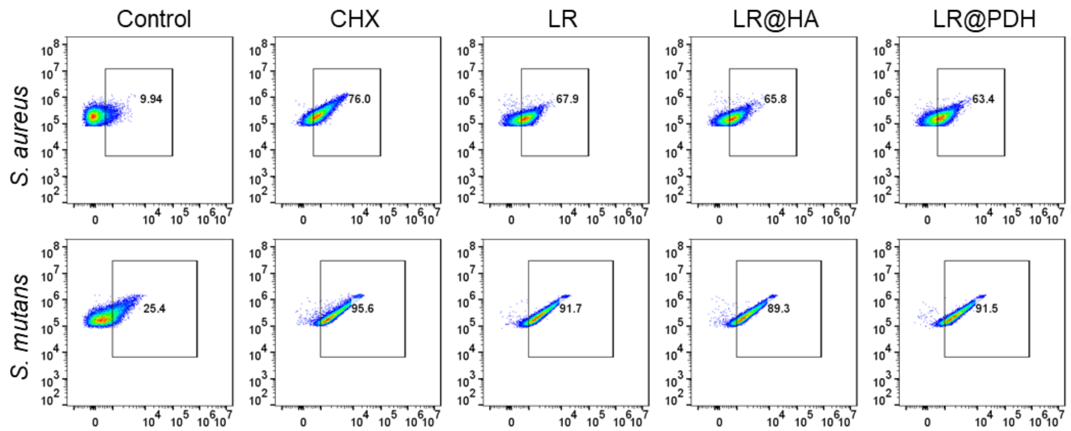

Fig. S13 Flow cytometry evaluated the antibacterial effects of CFS derived from LR, LR@HA, and LR@PDH via propidium iodide (PI) staining against *S. aureus* and *S. mutans*.

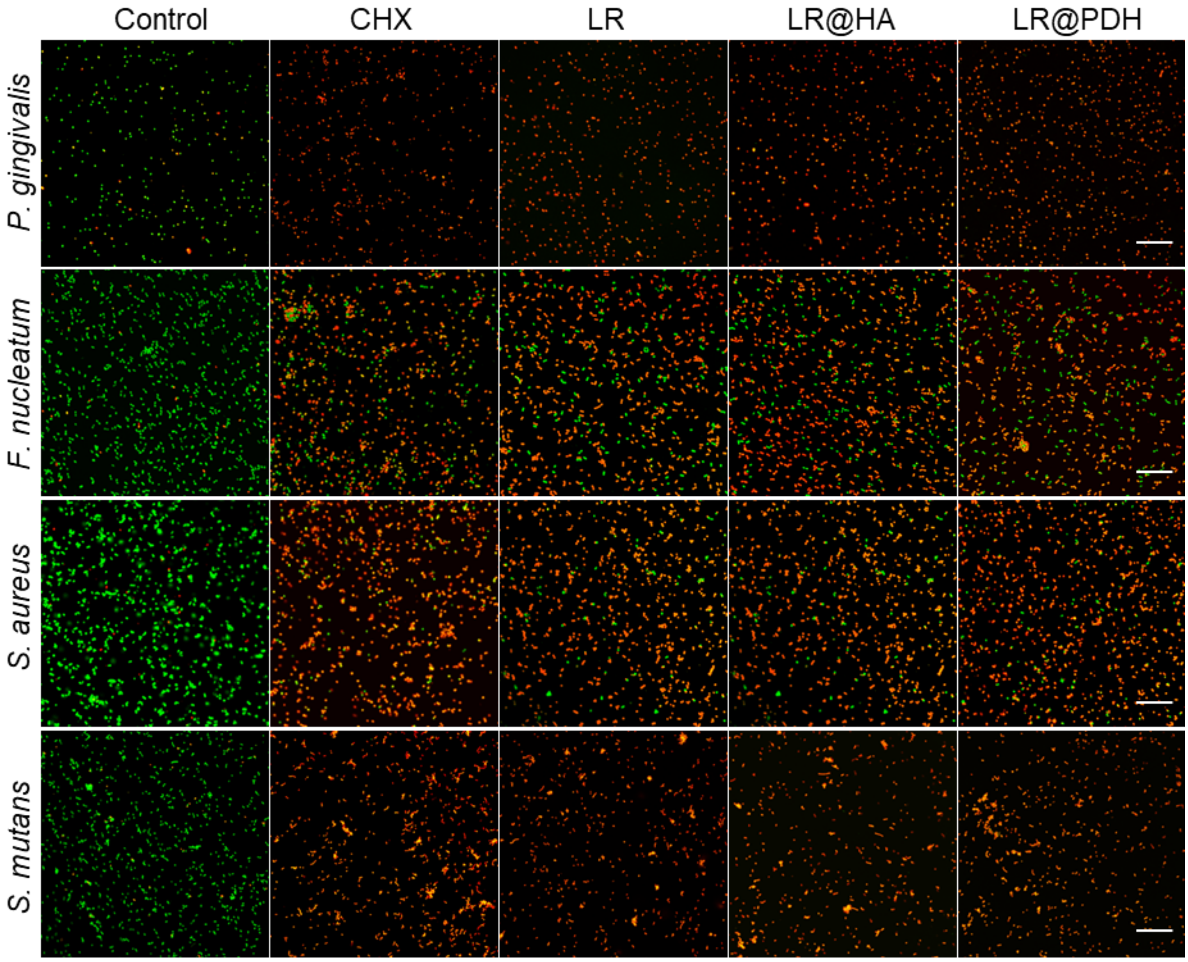

Fig. S14. CLSM of live/dead staining of *P. gingivalis, F. nucleatum, S. aureus and S. mutans* after treated with MRS, chlorhexidine, and LR, LR@HA, and LR@PDH-derived CFS. Scale bar, 20 μm.

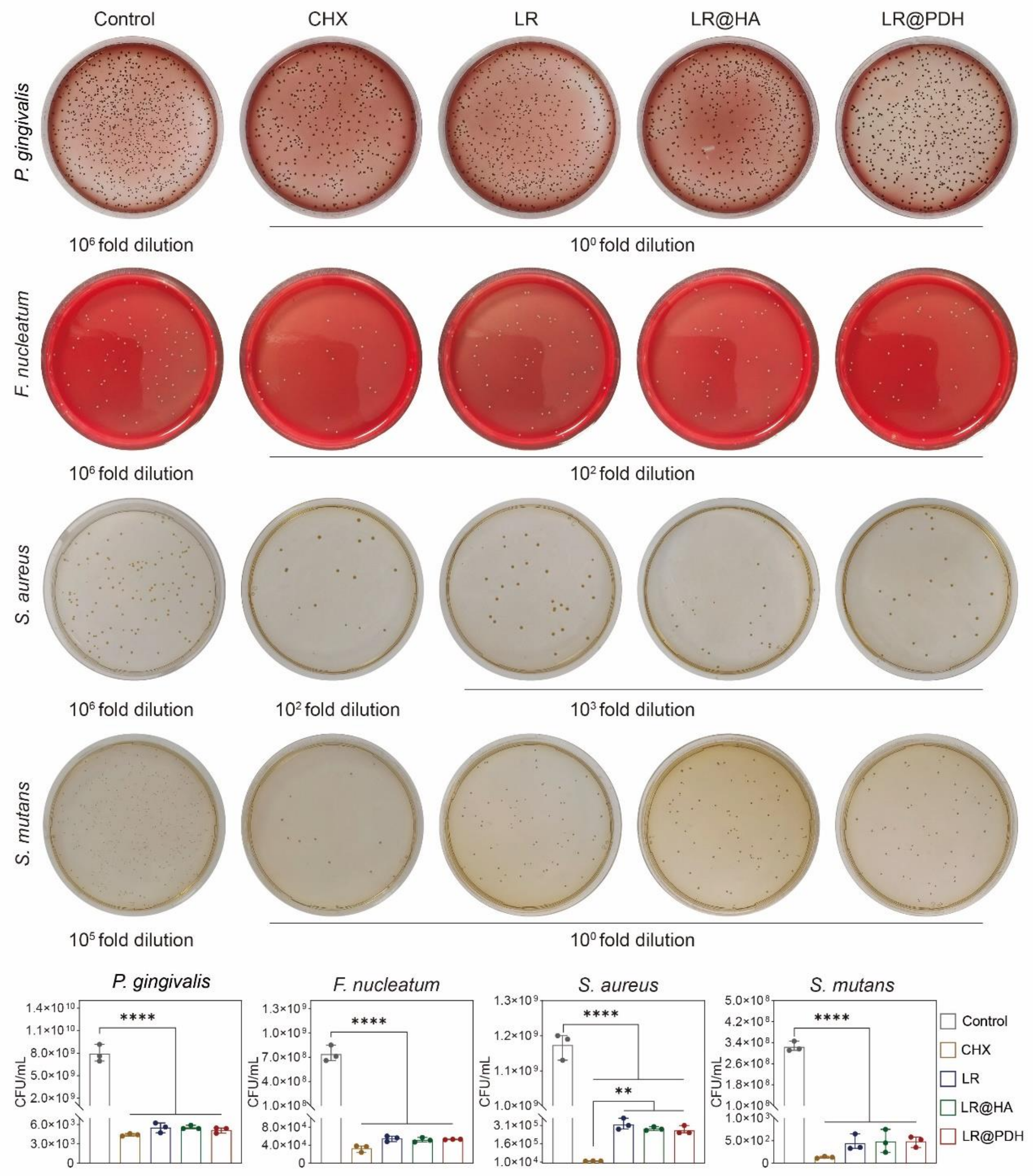

Fig. S15 Representative photographs and CFU counts of colonies of *P. gingivalis, F. nucleatum, S. aureus and S. mutans* on agar plates after treatment with MRS, chlorhexidine, and LR, LR@HA, and LR@PDH-derived CFS by the spread plate method. $^{**}p < 0.01$, $^{****}p < 0.0001$. The data are presented as mean ±SD, n = 3.

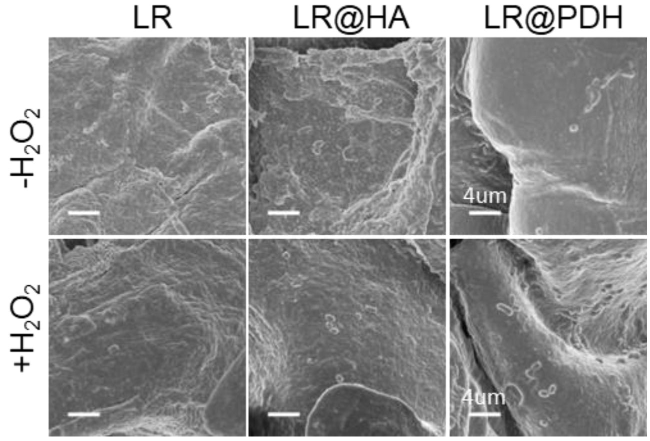

Figure S16 SEM images of oral tissues surface treated with LR, LR@HA and LR@PDH with or without H_2O_2 condition.

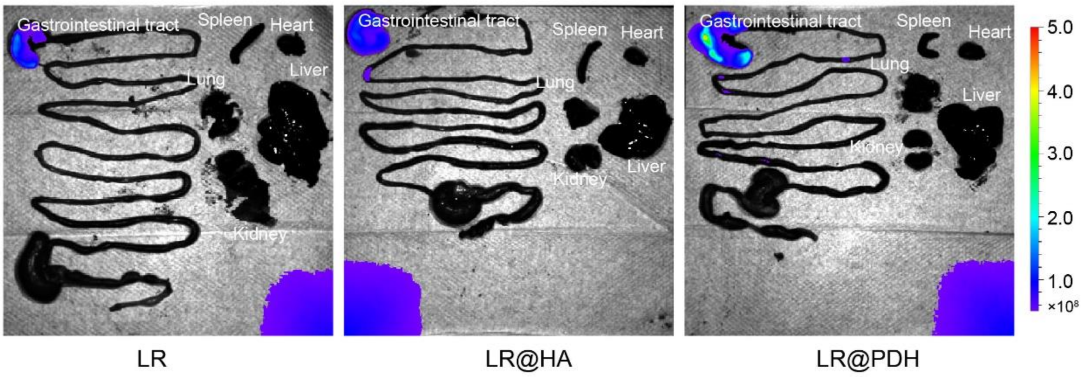

Fig. S17. Fluorescence images of the intestines and other major organs (heart, liver, spleen, lung and kidney) of rats after treatment with DIR-labeled LR, LR@HA, and LR@PDH for 24h.

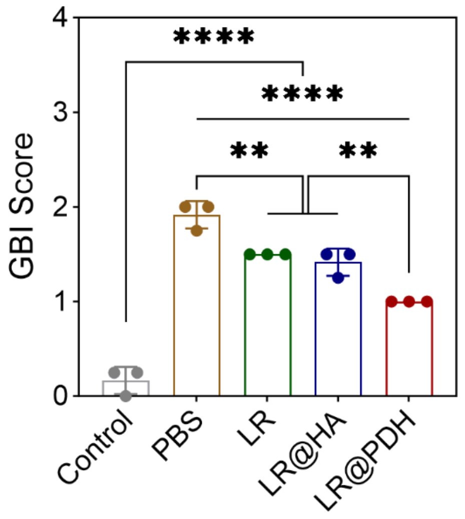

Figure S18 Assessment of the gingival bleeding index (GBI) score after various treatments. **$p < 0.01$, ****$p < 0.0001$. The data are presented as mean ±SD, n = 3.

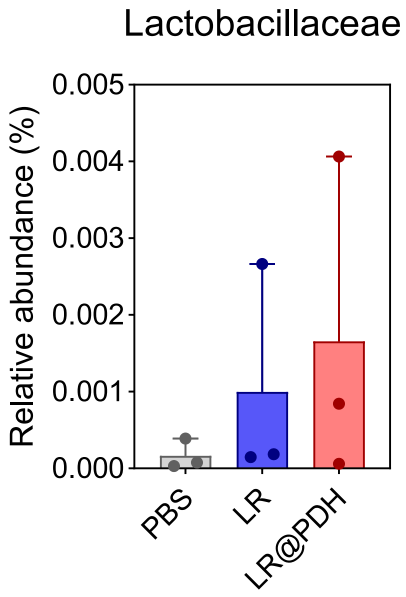

Fig. S19. Relative abundance of Lactobacillaceae in subgingival microbiome of different groups. The data are presented as mean ±SD, n = 3.

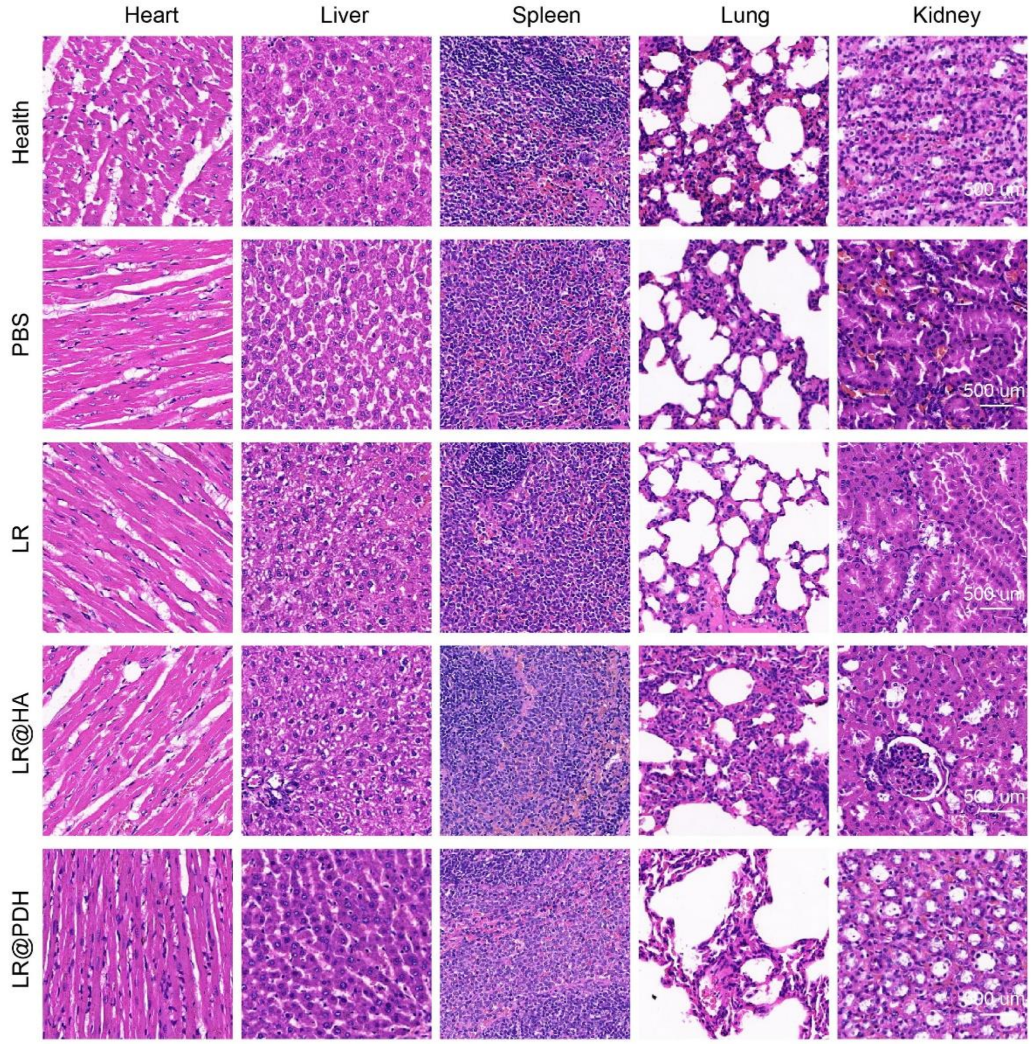

Fig. S20. Images of major organs (heart, liver, spleen, lung, kidney) stained by H&E in periodontitis rats after different treatments. Scale bar, 500 μm.

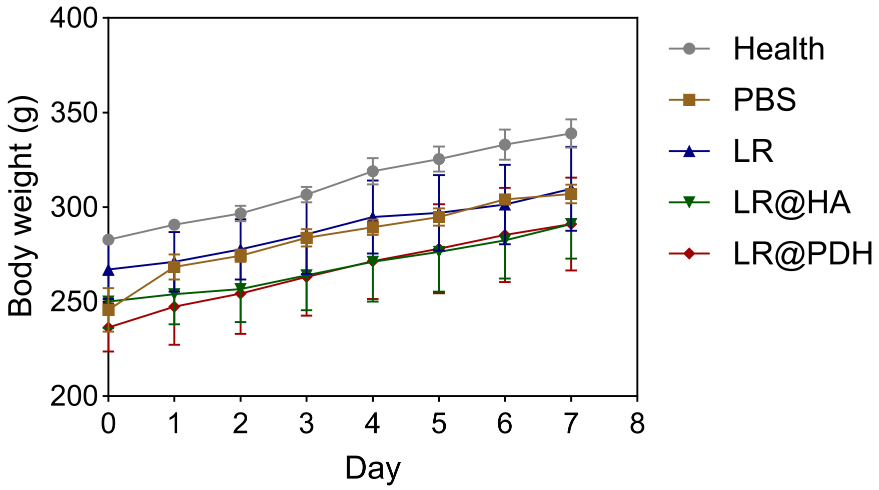

Fig. S21. Rat body weight during the experimental period. The data are presented as mean ±SD, n = 3.

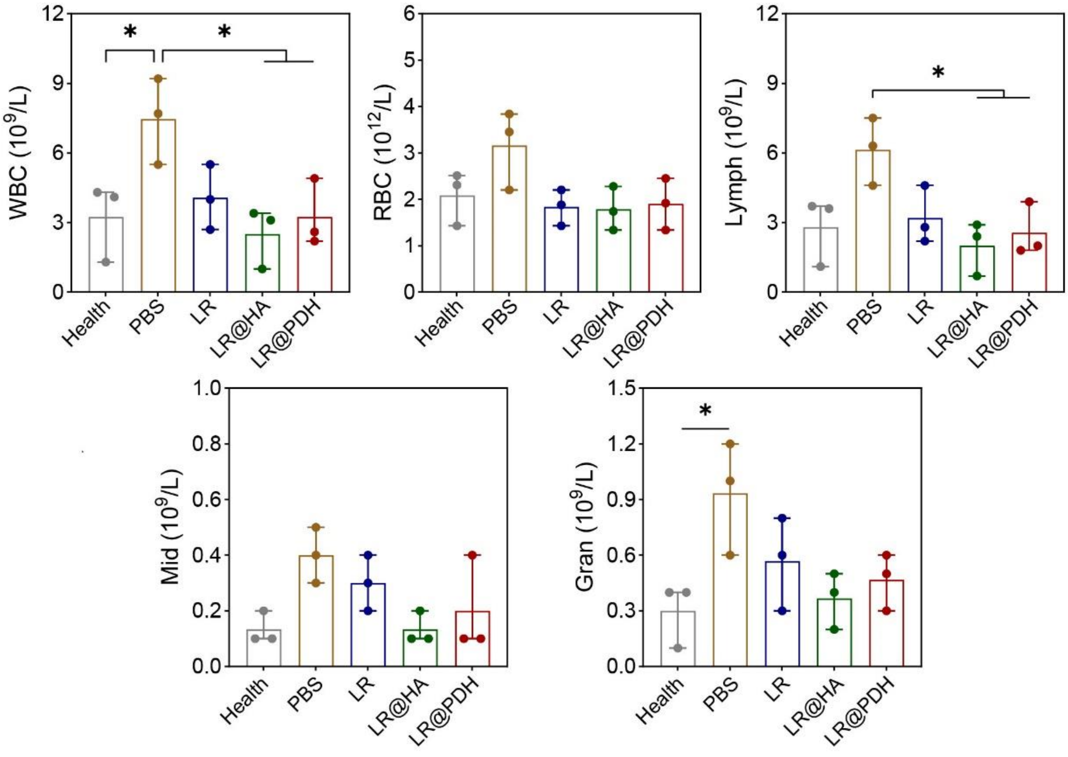

Fig. S22. Results of routine blood examination at the experimental endpoint. WBC, white blood cells; RBC, red blood cells; Lymph, lymphocytes; Mid, intermediate cells; Gran, neutrophile granulocyte. *$p < 0.05$. The data are presented as mean ±SD, n = 3.

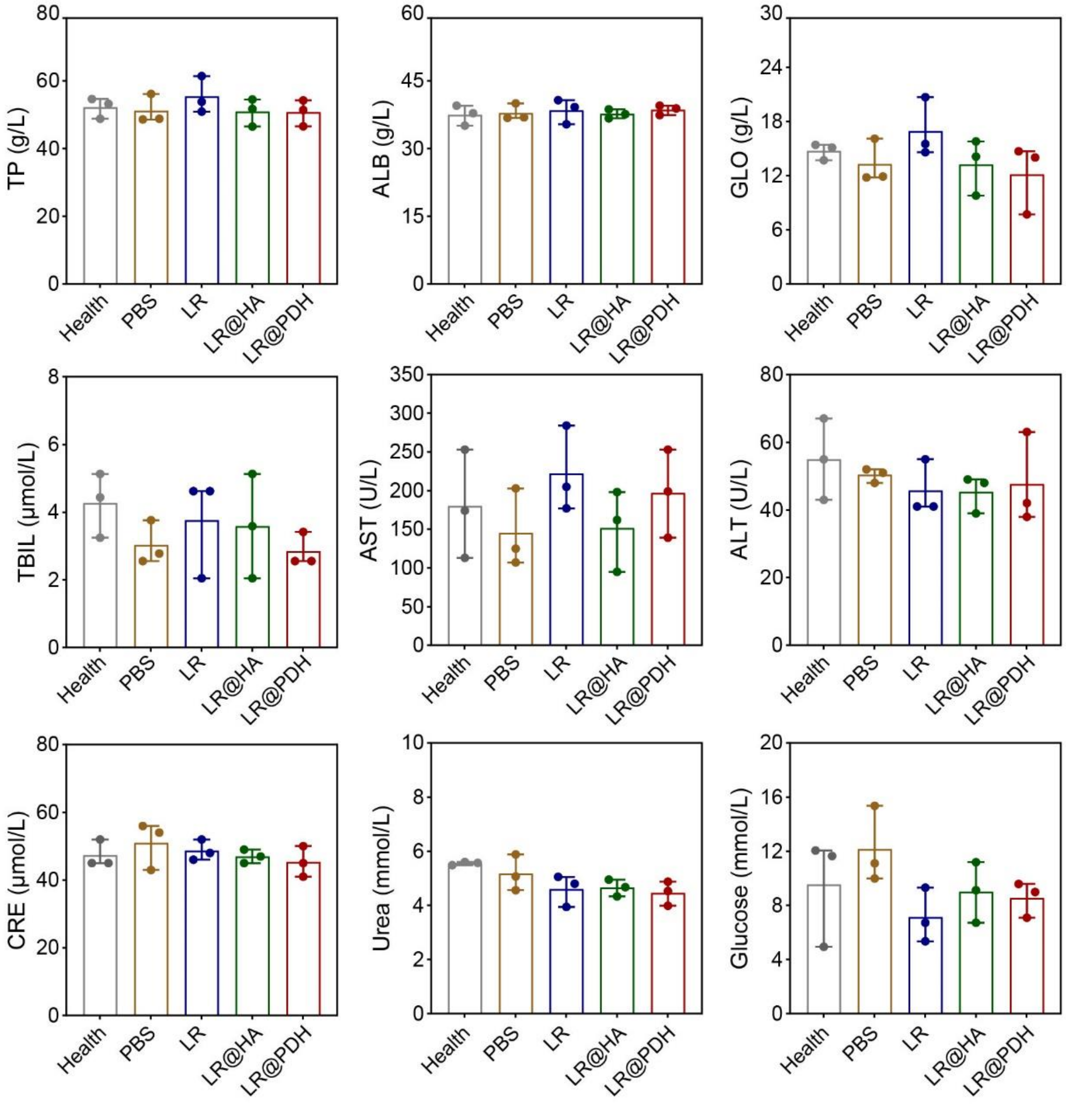

Fig. S23. Results of key serological analyses at the experimental endpoint. TP, total proteins; ALB, albumin; GLO, globulin; TBIL, total bilirubin; AST, aspartate aminotransferase; ALT, alanine aminotransferase; CRE, creatinine. The data are presented as mean ±SD, n = 3.
